# High-Definition Analysis of Host Protein Stability during Human Cytomegalovirus Infection Reveals Antiviral Factors and Viral Evasion Mechanisms

**DOI:** 10.1016/j.chom.2018.07.011

**Published:** 2018-09-12

**Authors:** Katie Nightingale, Kai-Min Lin, Benjamin J. Ravenhill, Colin Davies, Luis Nobre, Ceri A. Fielding, Eva Ruckova, Alice Fletcher-Etherington, Lior Soday, Hester Nichols, Daniel Sugrue, Eddie C.Y. Wang, Pablo Moreno, Yagnesh Umrania, Edward L. Huttlin, Robin Antrobus, Andrew J. Davison, Gavin W.G. Wilkinson, Richard J. Stanton, Peter Tomasec, Michael P. Weekes

**Affiliations:** 1 Cambridge Institute for Medical Research, University of Cambridge, Hills Road, Cambridge CB2 0XY, UK; 2 Cardiff University School of Medicine, Division of Infection and Immunity, Henry Wellcome Building, Heath Park, Cardiff CF14 4XN, UK; 3 Regional Centre for Applied Molecular Oncology (RECAMO), Masaryk Memorial Cancer Institute, Zluty Kopec 7, 65653 Brno, Czech Republic; 4 MRC-University of Glasgow Centre for Virus Research, Sir Michael Stoker Building, 464 Bearsden Road, Glasgow G61 1QH, UK; 5 Department of Cell Biology, Harvard Medical School, 240 Longwood Avenue, Boston, MA 02115, USA

**Keywords:** quantitative proteomics, tandem mass tag, pulsed SILAC, host-pathogen interaction, immune evasion, innate immunity, restriction factor, proteasome, lysosome, protein degradation

## Abstract

Human cytomegalovirus (HCMV) is an important pathogen with multiple immune evasion strategies, including virally facilitated degradation of host antiviral restriction factors. Here, we describe a multiplexed approach to discover proteins with innate immune function on the basis of active degradation by the proteasome or lysosome during early-phase HCMV infection. Using three orthogonal proteomic/transcriptomic screens to quantify protein degradation, with high confidence we identified 35 proteins enriched in antiviral restriction factors. A final screen employed a comprehensive panel of viral mutants to predict viral genes that target >250 human proteins. This approach revealed that helicase-like transcription factor (HLTF), a DNA helicase important in DNA repair, potently inhibits early viral gene expression but is rapidly degraded during infection. The functionally unknown HCMV protein UL145 facilitates HLTF degradation by recruiting the Cullin4 E3 ligase complex. Our approach and data will enable further identifications of innate pathways targeted by HCMV and other viruses.

## Highlights

• Multiplexed viral screens uncover host proteins targeted by early HCMV infection
• Finding host proteins targeted for degradation by HCMV reveals immune evasion strategies
• A screen of HCMV deletion mutants discovers viral factors that target >250 host proteins
• HLTF is an antiviral restriction factor that is targeted for degradation by HCMV UL145

## Graphical Abstract

**Figure:**
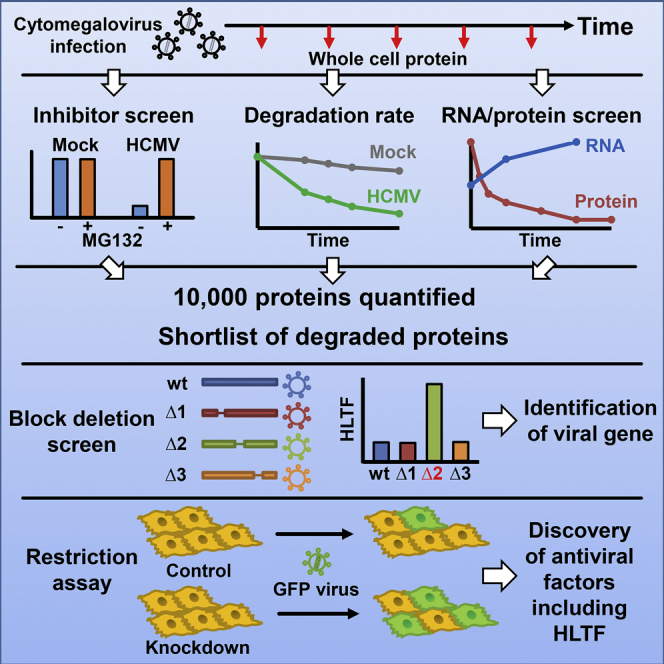

## Teaser

Nightingale et al. describe a multiplexed proteomic approach to discover antiviral restriction factors based on their degradation during human cytomegalovirus (HCMV) infection. Helicase-like transcription factor, an antiviral factor, is targeted by host Cullin4 E3 ligase complex for rapid degradation. HCMV UL145 hijacks Cullin4 to invoke this immune evading strategy.

## Introduction

Human cytomegalovirus (HCMV) is a ubiquitous herpesvirus that persistently infects the majority of the world's population (Mocarski et al., 2013). Following primary infection, HCMV establishes a lifelong latent infection under the control of a healthy immune system (Reeves et al., 2005). Reactivation from viral latency to productive infection causes serious disease in immunocompromised individuals, particularly transplant recipients and AIDS patients (Nichols et al., 2002). Primary infection and reactivation *in utero* are leading causes of deafness and mental retardation in newborns, affecting approximately 1 in 200 pregnancies (Mocarski et al., 2013).

Susceptibility to viral infection and disease is determined in part by antiviral restriction factors (ARFs) and the viral proteins that have evolved to degrade them (Duggal and Emerman, 2012). Small-molecule disruption of the interaction between an ARF and a viral antagonist can enable endogenous inhibition of viral replication (Nathans et al., 2008). The identification and characterization of ARFs therefore has important implications for antiviral therapy, and is particularly important for HCMV, for which only a few drugs are available.

HCMV is a paradigm for viral immune evasion, encoding at least 14 proteins that inhibit natural killer (NK) or T cell activation. A common final pathway for many host protein targets is proteasomal or lysosomal degradation (reviewed in Halenius et al., 2015). HCMV also modulates intrinsic immunity to facilitate viral replication, degrading components of cellular promyelocytic leukemia nuclear bodies (PML-NB) Sp100, MORC3, and DAXX that act as restriction factors (Kim et al., 2011, Schreiner and Wodrich, 2013, Sloan et al., 2016, Tavalai et al., 2011). We previously published a systematic temporal analysis that detailed how HCMV orchestrates the expression of >8,000 cellular proteins over the whole course of infection (Weekes et al., 2014). However, >900 proteins were downregulated >3-fold, making challenging the prediction of which molecules are most likely to perform functions in adaptive and innate immunity. Similarly high numbers of protein targets have subsequently been observed in systematic studies of infections by other viruses, for example Epstein-Barr virus (Ersing et al., 2017) and HIV (Matheson et al., 2015).

Here, we describe a multiplexed proteomic approach to identify molecules of key functional importance in innate immunity, on the basis of their active proteasomal or lysosomal degradation during the early phase of HCMV infection. We employ three orthogonal tandem mass tag (TMT)-based proteomic screens to measure protein degradation. The first measures protein abundance throughout early infection in the presence or absence of inhibitors of the proteasome or lysosome. The second employs an unbiased global pulse-chase to compare the rates of protein degradation during HCMV with mock infection. The third compares transcript and protein abundance over time to distinguish between degraded and transcriptionally regulated proteins. Our data provide a comprehensive analysis of protein degradation and synthesis during early viral infection, revealing how and when HCMV regulates the expression of >10,000 host proteins and their transcripts to facilitate replication and immune evasion.

During productive infection *in vitro*, HCMV gene expression is conventionally divided into immediate-early, early, and late phases over a replication cycle lasting ∼96 hr. Further definition can be gained by measuring viral protein profiles over time, which we have used previously to define five temporal classes of viral protein expression (Weekes et al., 2014). All herpesviruses have large genomes, potentially encoding hundreds of open reading frames (ORFs) (Davison et al., 2013, Stern-Ginossar et al., 2012), meaning that identification of which individual gene targets a given cellular factor can be challenging. To facilitate the mapping of viral gene functions we employed a panel of HCMV mutants, each deleted in contiguous gene blocks dispensable for virus replication *in vitro*. A systematic proteomic screen of these mutants defined the genetic loci responsible for targeting >250 host proteins.

A key biological insight from our data is the prediction of ARFs. The RING E3 ligase helicase-like transcription factor (HLTF) was proteasomally degraded throughout early infection and potently inhibits viral immediate-early gene expression. HLTF was found to be targeted by a protein encoded by the U_L_/*b*′ region in the HCMV genome (UL133-UL150). Among the proteins encoded by this region, UL145, which previously had no known function, was necessary and sufficient for HLTF degradation via recruitment of the Cullin 4/DDB1 ligase complex. Our approach and data predict molecules of importance in innate antiviral immunity and will enable further identifications of host pathways targeted by viruses.

## Results

### Host Proteins Targeted for Degradation Early during HCMV Infection

**Figure 1:**
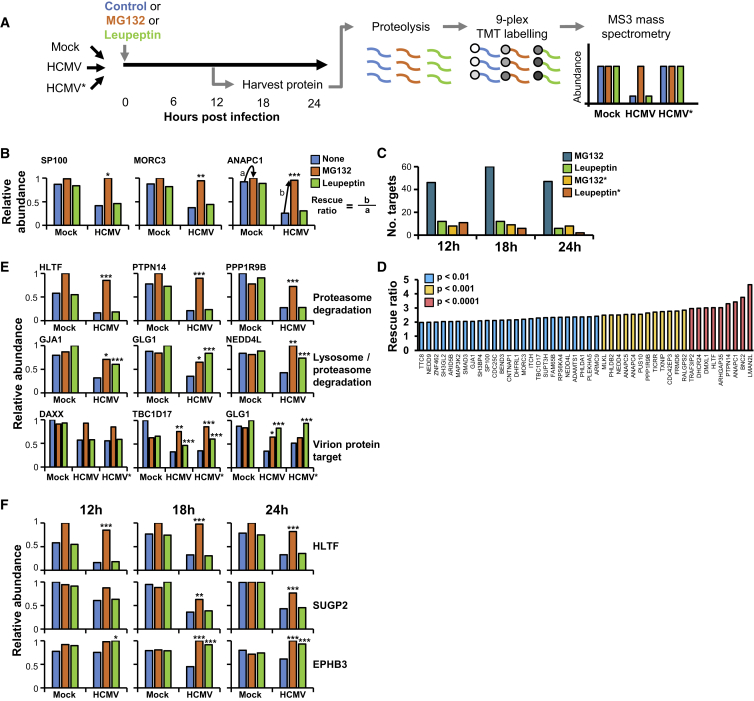
Identification of Proteins Targeted for Proteasomal or Lysosomal Degradation by HCMV Using an Inhibitor-Based Proteomic Screen (A) Schematic of the experimental workflow. Three similar experiments were conducted, examining 12, 18, and 24 hr of HCMV infection; the workflow illustrates the 12-hr analysis. The HFFF-TERT cells used for this analysis behaved extremely similarly to primary HFFFs upon infection with HCMV (Figure S1D). A comparison of two different protocols for “mock” infection suggested that no adventitious factors were carried along in our viral preparations (Figure S1E). (B) Examples of positive controls from the existing literature that were validated by this screen. A “rescue ratio” was calculated as shown: (protein abundance during HCMV infection with inhibitor/abundance during infection without inhibitor) [b]/(protein abundance during mock infection with inhibitor/abundance without inhibitor) [a]. [a] was limited to a minimum of 1 to avoid artificial ratio inflation. As these ratios were approximately normally distributed (Figure S1B), Benjamini-Hochberg adjusted significance A values were used to estimate p values (see STAR Methods). ^∗^p < 0.05, ^∗∗^p < 0.005, ^∗∗∗^p < 0.0005 are shown above the bars for HCMV + MG132 or HCMV + leupeptin. Further examples are shown in Figure S2A. (C) Number of proteins rescued by MG132 or leupeptin at each time point studied. A stringent filter was employed that required >1.5-fold downregulation during infection with HCMV or irradiated HCMV, and a rescue ratio of >1.5 with an associated p value of <0.01 (all criteria for each experiment are described in Figure S1C). (D) Rescue ratios of all 46 proteins identified by the 12-hr MG132 screen, using stringent criteria. (E) Examples of degraded proteins identified using MG132 (top panels), leupeptin and MG132 (middle panels), or irradiated HCMV plus either inhibitor (bottom panels). Further examples are shown in Figure S2B. Color coding for bars and rescue ratio p values are as described in (B). (F) Examples of proteins that were degraded throughout the time course studied (HLTF), or restricted to a more limited period of infection (SUGP2 and EPHB3). Color coding for bars and rescue ratio p values are as described in (B).

To build a detailed global picture of all host proteins that are degraded during early HCMV infection, we applied the proteasomal inhibitor MG132 or the lysosomal protease inhibitor leupeptin at three early time points during infection of immortalized primary human fetal foreskin fibroblasts (HFFF-TERTs). Virus inactivated by irradiation (HCMV^∗^) was included in the experiment to determine whether components of the virion delivered during the process of infection made a contribution. MG132 is known to affect lysosomal cathepsins in addition to the proteasome (Wiertz et al., 1996), and leupeptin is a naturally occurring protease inhibitor that can inhibit some proteasomal proteases in addition to the lysosome. Our intention in using these broad, well-characterized inhibitors was to obtain a comprehensive list of proteins targeted for degradation by HCMV, rather than deciphering whether a given protein was degraded in the proteasome or the lysosome. TMT peptide labels and MS3 mass spectrometry enabled very precise protein quantitation, as well as multiplexed analysis of up to 11 samples in the same experiment (Figure 1A) (McAlister et al., 2014).

We quantified 8,118–8,678 proteins (Figure S1A), and determined an MG132 and leupeptin “rescue ratio” for each protein, obtained by comparing protein abundance during HCMV infection ± inhibitor with protein abundance during mock infection ± inhibitor (Figure 1B). This ratio enabled identification of proteins that exhibited increased degradation during HCMV infection, as opposed to those having a baseline high turnover in mock-infected cells. Using stringent criteria (Figure S1C), data were filtered to identify proteins that were most strongly downregulated by HCMV and most significantly rescued by the inhibitor. Overall, 131 proteins were rescued by application of MG132 within 24 hr of infection, with 46 proteins rescued at 12 hr post infection, the earliest time point studied (Figure 1C). Of the 46 proteins, 7 have already been reported to be degraded by HCMV, including HCMV restriction factors Sp100 and MORC3 (Kim et al., 2011, Sloan et al., 2016, Tavalai et al., 2011), E3 ubiquitin ligases ANAPC1, 4, and 5 (anaphase promoting complex subunits 1, 4, and 5) and ITCH (itchy E3 ubiquitin protein ligase) (Figures 1B and S2A) (reviewed in Weekes et al., 2014). The remaining 39 proteins have not previously been reported to be targeted for proteasomal degradation by HCMV, including HLTF (Figures 1D and 1E).

Overall, 28 proteins were rescued by application of leupeptin, of which 50% were also rescued by MG132 (Figures 1C, 1E, and S2B). Of these proteins, 12 were rescued at 12 hr post infection, including connexin family gap junction protein alpha 1 (GJA1), which has previously been reported to be degraded during HCMV infection (Stanton et al., 2007). Among other findings, we now report early rescue of E3 ligases neural precursor cell expressed, developmentally downregulated 4 (NEDD4) and NEDD4-like (NEDD4L). Some proteins were degraded throughout early infection, whereas others including ephrin receptor B3 (EPHB3) were most significantly degraded during a more limited interval, which may reflect the kinetics of expression of the HCMV proteins that target them (Weekes et al., 2014) (Figures 1F and S2B).

During infection with irradiated HCMV, application of inhibitors resulted in rescue of 37 proteins (Figure 1C). These included the HCMV restriction factor DAXX, which is known to be targeted for degradation by the viral tegument protein pp71 (Schreiner and Wodrich, 2013). Additional proteins targeted by virion components included the fibroblast growth factor receptor Golgi glycoprotein 1 (GLG1), which has not previously been reported to play a role in innate immunity (Figure 1E).

Data from all proteomic experiments in this study are shown in Table S1. Here, the worksheet “Plots” is interactive, enabling generation of graphs of protein expression of any of the human and viral proteins quantified. Table S2 shows lists of proteins identified by each screen.

### Stability of Viral Proteins

To identify as many HCMV proteins as possible, we used a protein database that included 170 canonical ORFs most likely to encode functional proteins, 604 non-canonical ORFs identified as potentially protein-coding by ribosome profiling (Stern-Ginossar et al., 2012), and all ORFs of ≥8 amino acids from a six-frame translation of the HCMV strain Merlin sequence. This analysis identified expression of 139 of 170 canonical ORFs, 27 of 604 non-canonical ORFs, and 13 ORFs from the six-frame translation (6FT-ORFs) that had not previously been recognized, some of these from multiple peptides. Of the 13 6FT-ORFs, 11 were encoded in alternative reading frames from canonical ORFs, and 2 represented 5′-terminal extensions of previously described ORFs (one of canonical US20 and the other of non-canonical ORFL147C) (Figure S1A and Data S1).

The application of MG132 during infection led to substantial changes in the abundance of a number of viral proteins, particularly at 18 hr and 24 hr post infection. The most substantially rescued proteins included non-canonical ORFs or 6FT-ORFs. Leupeptin led to less substantial, but nevertheless significant changes (Figure S2C). One possible explanation may be that a subset of non-canonical ORFs represents rapidly degraded translation “noise,” encoding proteins that are likely to be unstructured and inherently unstable. This hypothesis is consistent with a comparison of the disposition of the 13 6FT-ORFs in the genome sequences of 244 HCMV strains, which suggested that at least 12 are unlikely to encode functional proteins (Data S1). Another possibility is that certain viral proteins are rapidly co-degraded with human target proteins. We have previously reported that HCMV UL138 is co-degraded in the lysosome with the multi-drug transporter ABCC1 (Weekes et al., 2013) and that the ten US12-US21 proteins target certain cell-surface proteins for lysosomal degradation (Fielding et al., 2017). UL138 and 5 of the 5 quantified US12-US21 proteins were all substantially rescued by leupeptin (Figure S2C), suggesting that the group of viral proteins that exhibit the greatest rescue by inhibitors may be enriched in molecules that regulate important host targets.

### Global Overview of Protein Synthesis and Degradation during Infection in an Unbiased Pulsed SILAC/TMT Screen

**Figure 2:**
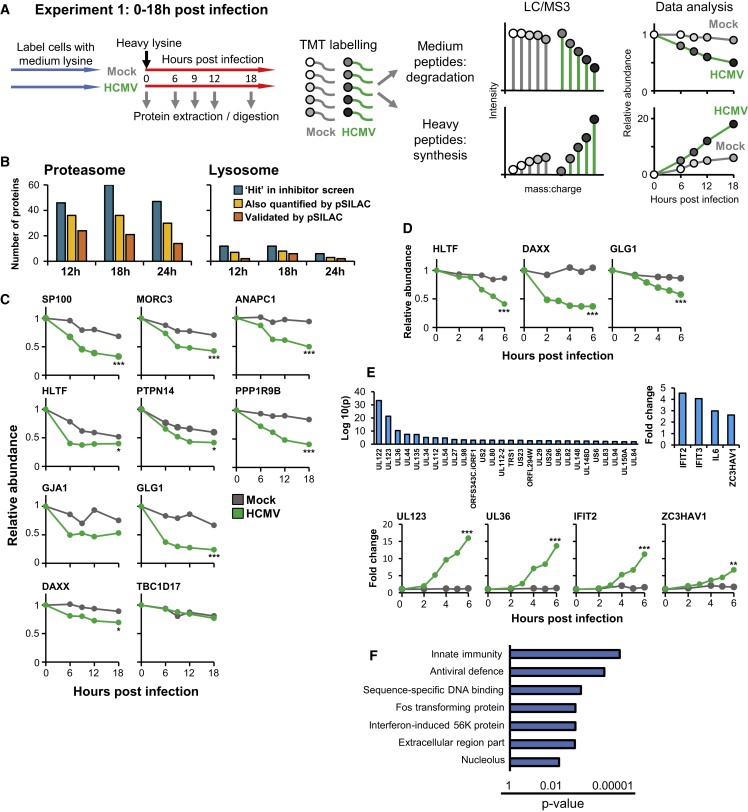
A pSILAC/TMT-Based Screen to Quantify Rates of Protein Degradation and Synthesis (A) Schematic of the experimental workflow. Experiment 1 is illustrated; the equivalent Experiment 2 instead included time points 0, 2, 3, 4, 5, and 6 hr after infection. We calculated a p value for the difference between Kdeg_HCMV_ and Kdeg_mock_ as described in STAR Methods (Statistical Analysis). If Kdeg_mock_ was >0, a fold change (FC_HCMV_) in protein abundance in the HCMV-infected sample at 18 hr (Experiment 1) or 6 hr (Experiment 2) was calculated, compared with time point 0 (see also Figure S1C). (B) Overlap between the inhibitor and pSILAC screens. Blue bars show the number of proteins rescued by MG132 or leupeptin at each time point (Figure 1C). A given protein was considered to be quantified by pSILAC if measured in either the 6-hr or 18-hr screen. pSILAC data were considered to be consistent with the inhibitor data if Kdeg_HCMV_/Kdeg_mock_ > 1.5 or FC_HCMV_ > 1.5 (sensitive criteria, Figure S1C). (C) 18-hr pSILAC validation of positive controls and targets identified by the inhibitor screen (Figures 1B and 1E). NEDD4L was not quantified in this experiment. (D) Examples of 6-hr pSILAC data for proteins degraded very early during HCMV infection. (E) Twenty-eight viral and four human proteins synthesized to significantly greater levels in HCMV-infected cells compared with mock-infected cells from the 0- to 6-hr pSILAC analysis (filters and p-value calculations described in STAR Methods [Statistical Analysis]). Example plots are shown in the lower part of the figure. (F) Enrichment of pathways within human proteins synthesized at significant levels during the 0- to 18-hr analysis, using DAVID software (see also Table S3C). Filters were as described in (E). For (C), (D), and (E), ^∗^p < 0.05, ^∗∗^p < 0.005, and ^∗∗∗^p < 0.0005.

To address protein stability and turnover using an orthogonal approach, we combined pulsed SILAC (stable isotope labeling with amino acids in cell culture) (pSILAC) with TMT to compare the rates of protein degradation during HCMV and mock infection (degradation rate constants: Kdeg_HCMV_, Kdeg_mock_). Compared with SILAC-only experiments, benefits of this multiplexed approach were a dramatic reduction in the amount of mass spectrometry time required, and the measurement of each protein at every time point, avoiding problems caused by proteins being quantified in some but not all samples. Two screens examined the first 6 hr or first 18 hr of infection (Figures 2A and S3A). Of the proteins degraded at one or more time points in the inhibitor screen, 49% (MG132) and 38% (leupeptin) exhibited an increased rate of degradation by pSILAC (Figures 2B, 2C, S2A, and S2B). In some cases, proteins were degraded extremely early during infection. For example, a significant difference was observed between HCMV and mock infection within 4 hr for HLTF, DAXX, and GLG1 (Figure 2D). Degradation of the restriction factor DAXX has been shown to play a vital role in activation of immediate-early HCMV gene expression (reviewed in Schreiner and Wodrich, 2013). Similarly prompt degradation of HLTF and GLG1 suggests that these proteins may play an important role in the early part of the viral life cycle.

Of 46 proteins rescued at 12 hr with MG132, 36 were quantified in pSILAC and 24 of 36 exhibited increased degradation in HCMV-infected cells compared with mock infection. We investigated 12 out of 36 proteins that were rescued at 12 hr with MG132 and yet did not exhibit instability by pSILAC. In 3 of 12 cases, the protein did not start to exhibit increased degradation during the 6-hr pSILAC screen and was not quantified in the 18-hr analysis (for example, NEDD4, Figure S2B). In 2 of 12 cases, Kdeg_HCMV_/Kdeg_mock_ or FC_CMV_ was >1 but <1.5 (for example, PHLDB2, Figure S3B). Thioredoxin-interacting protein (TXNIP) was extremely rapidly turned over during mock infection, making it difficult to assess a difference between mock and HCMV infection even during the 6-hr pulse. Pleckstrin homology-like domain family A member 1 (PHLDA1) was rescued significantly by the application of MG132 during mock infection, and this may have made the rescue ratio (which is a ratio of ratios) less precise. Overall, at least 50% of proteins that were rescued by MG132 but did not exhibit instability by pSILAC may nevertheless be degraded, and in some cases transcriptional downregulation and post-translational controls may have worked in combination (Figure S3B).

pSILAC enabled the rate of synthesis of each protein in HCMV-infected (Ksyn_HCMV_) and mock-infected cells (Ksyn_mock_) to be compared. It was also possible to distinguish heavy-labeled proteins synthesized after infection from light-labeled proteins delivered in the viral particle. In the 6 hr pulse-chase, 28 viral and 4 human proteins were synthesized to significantly greater levels in HCMV-infected cells compared with mock-infected cells; all 4 human proteins are known to be interferon responsive (Figure 2E) (Rusinova et al., 2013). By 18 hr of pulse-chase, 72 viral and 64 human proteins were synthesized at significantly greater levels (Tables S3A and S3B). Application of DAVID software (Huang da et al., 2009) to determine which pathways were enriched among these proteins indicated the upregulation of multiple pro-apoptotic transcription factors, complement components important in innate immunity, and known ARFs including MX1, MX2, DDX58, and ZC3HAV1. These proteins are likely to represent the components of an early cellular response to viral infection (Figure 2F and Table S3C).

### Transcriptional and Post-transcriptional Regulation of Expression

**Figure 3:**
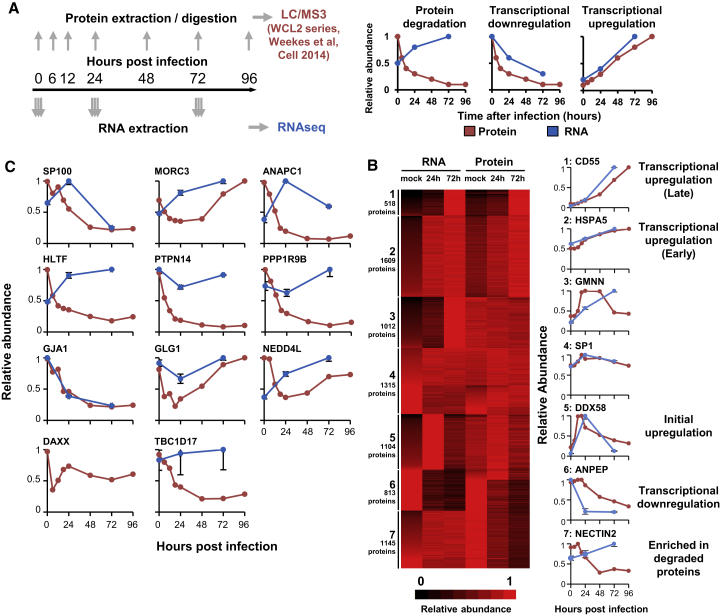
Comparative Analysis of Transcript and Protein Abundance to Identify the Mechanism of Host Protein Regulation (A) Schematic of the experimental workflow. (B) K-means-based hierarchical cluster analysis of 7,516 proteins and transcripts, identifying global mechanisms of protein regulation by HCMV. Right panels show examples of each class. (C) Examples of data for proteins shown in Figures 1B, 1E, and 2C. DAXX was not well quantified in RNA-seq.

To identify where protein expression was determined primarily by mRNA levels rather than being regulated at a post-transcriptional level, we compared transcript and protein abundance over time. When downregulation of a given protein was accompanied by transcript upregulation, it is likely that the protein was degraded. Thus, integration of an RNA/protein dataset with the other screens would identify the proteins that were degraded during infection, in addition to providing a global analysis of how viral infection regulates the host proteome and transcriptome. We published previously a temporal analysis of >8,000 proteins over eight time points spanning the course of infection (Weekes et al., 2014). We now compared data for human proteins with RNA sequencing (RNA-seq) analysis from samples derived from simultaneous infections and harvests (Figure 3A).

The k-means method is useful for clustering proteins into a specified number of classes based on the similarity of kinetic expression profiles. K-means clustering with 1–20 classes suggested there were at least seven different patterns of expression of RNA and protein (Figures 3B and S4A; Table S2). Clusters 1 and 2 chiefly comprised proteins that were transcriptionally upregulated, including CD55, which is known to be upregulated during infection. Cluster 5 included proteins that were initially upregulated at the level of transcription and then downregulated. Cluster 6 included transcriptionally downregulated proteins, including CD13/alanyl aminopeptidase (ANPEP). Cluster 7 was enriched in proteins known to be degraded during HCMV infection, including the NK-activating ligand CD112/nectin cell adhesion molecule 2 (NECTIN2) (reviewed in Weekes et al., 2014), as well as multiple “hits” from the inhibitor and pSILAC screens (Table S2).

Multiple proteins identified as degraded by other screens were also identified by the RNA/protein screen, including most of those shown in Figures 1B, 1E, S2A, and S2B. However, certain proteins were not identified, usually as a result of the stringent criteria applied (Figure S1C). For example, protein tyrosine phosphatase, non-receptor type 14 (PTPN14) protein was downregulated >12-fold during infection, but the transcript was downregulated 1.1- to 1.4-fold as opposed to being upregulated, which was a requirement of the screen (Figure 3C). Nevertheless, from these kinetics and the results of the other two screens it is likely that PTPN14 is degraded, suggesting that an overall shortlist of “high-confidence” degraded proteins should include those passing at least 2 out of 3 screening tests.

### Degradation of Multiple E3 Ubiquitin Ligases Early during HCMV Infection

**Figure 4:**
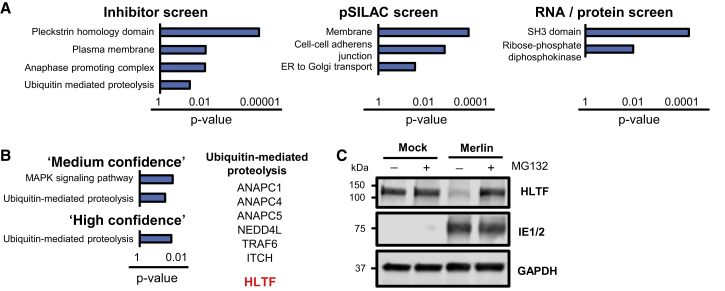
Complementarity between Individual Screens Identified a Shortlist of Proteins Enriched in Ubiquitin E3 Ligases, Including HLTF (A) DAVID analysis of pathway enrichment among proteins identified by each screen (stringent criteria, Figure S1C). Benjamini-Hochberg adjusted p values are shown for each pathway. See also STAR Methods (Pathway Analysis). (B) Enrichment of pathways using DAVID software as described in (A). (C) Immunoblot confirmed rescue of HLTF by MG132 (MOI = 5, 12-hr infection, MG132 applied from 0 to 12 hr).

We applied DAVID software (Huang da et al., 2009) to determine which pathways were enriched among degraded proteins from each individual screen. The results suggested that multiple plasma membrane proteins may be degraded during infection, in particular proteins that include a pleckstrin homology (PLEKH) domain (for example PLEKHA5) or that function in cell-to-cell adhesion (Figures 4A and S5A; Tables S4A–S4C). We found previously that HCMV rapidly downregulates multiple γ-protocadherins (PCDHGC). We now show that a subset of these proteins are degraded early during infection, including PCDHGB5, supporting the suggestion that these might be NK or T cell ligands or cellular receptors for HCMV (Figure S5A and Table S4B) (Weekes et al., 2014). We report that ANAPC2, in addition to ANAPC1, 4, and 5, is degraded early during HCMV infection, suggesting that inhibition of these proteins may be of particular importance in subverting the host cell-cycle machinery during infection.

To identify with highest confidence the proteins that are degraded during infection, we combined data from all three screens. A “medium-confidence” shortlist included a total of 133 proteins degraded in ≥1 out of 3 screens by stringent criteria and degraded in at least one other screen by sensitive criteria (Figure S1C and Table S2). A “high-confidence” shortlist included 35 proteins that were degraded in at least 2 out of 3 screens by stringent criteria, with 7 proteins degraded in all 3 screens (Figure 4B and Table S2). As expected, the majority of proteins in both shortlists appeared in cluster 7 from the RNA/protein analysis (Figures 3B and S5B).

“Ubiquitin-mediated proteolysis” was the only significantly enriched pathway within the “high-confidence” shortlist, and included 6 ubiquitin E3 ligases (Figure 4B, Tables S2 and S4E). A comprehensive search of all 35 “high-confidence” proteins for E3 ligase activity identified one additional ligase, HLTF. HLTF was degraded in all three screens, and throughout early infection starting from 4 hr (Figures 1F, 2D, and 3C), which was confirmed by immunoblot (Figure 4C). This suggested that HLTF might play a key functional role in early viral infection, possibly being degraded by the virus to evade antiviral restriction.

HLTF is known to participate in error-free post-replication DNA-damage tolerance by binding to nascent single-stranded DNA and ubiquitinating DNA replication processivity factor (PCNA) at the stalled replication fork. HLTF thereby facilitates fork regression and reconvenes DNA replication (Achar et al., 2011, Blastyak et al., 2010). Functional domains in HLTF include an RING E3 ligase domain close to the C terminus, an N-terminal DNA-binding HIRAN (HIP116 Rad5p N-terminal) domain, and ATPase/helicase domains (Achar et al., 2011). Two recent studies have suggested that HIV Vpr also degrades HLTF in a proteasome-dependent manner, by redirecting the Cullin 4/DCAF1 E3 ligase complex, although neither study demonstrated why this is of functional importance to HIV (Hrecka et al., 2016, Lahouassa et al., 2016).

### HCMV UL145 Is Necessary and Sufficient to Degrade HLTF

**Figure 5:**
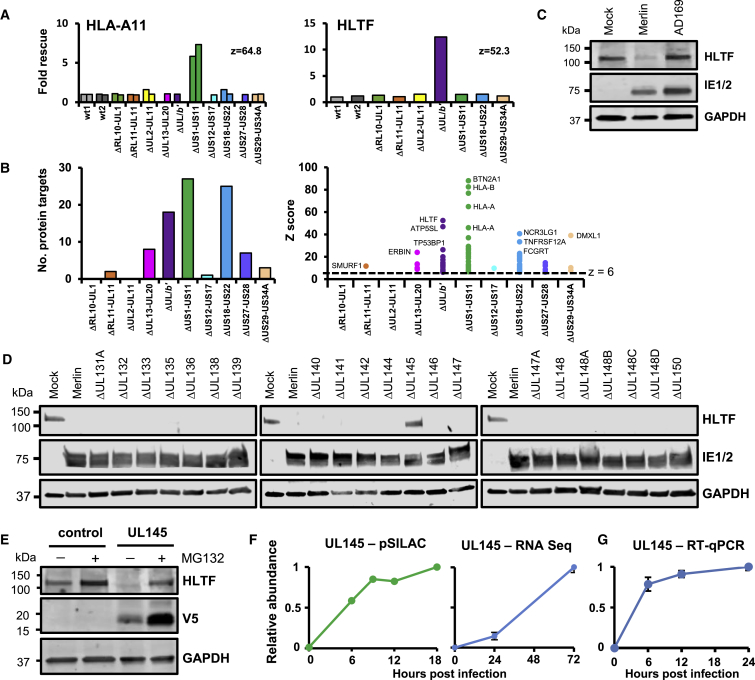
A Proteomic Screen of Viral Block Deletion Mutants Revealed that the U_L_/*b*′ gene UL145 Targets HLTF (A) Regulation of >250 cellular proteins (MOI = 10, 72-hr infection). Due to the multiplexing limits of TMT analysis, two separate screens were needed to encompass all viruses. HCMV strain AD169 was used for the U_L_/*b*′ mutant, as a deletion in this region (plus additional defects) has been acquired during passage in culture. Strain AD169, strain Merlin ΔUS27-US28, ΔUL13-UL20, and ΔUS12-US17 mutants were only examined in single screens, with all other viruses examined in duplicate. Example results are shown for HLA-A11 and HLTF. For HLTF, peptides were quantified in only one of the two screens. Further details are given in STAR Methods (Statistical Analysis). (B) Numbers of human proteins targeted by each block using stringent scoring (*Z* score of >6 and FC > 2, left panel). For each block, the *Z* scores of all proteins that passed the stringent scoring criteria are shown (right panel). (C) Immunoblot confirming that HLTF is downregulated by strain Merlin but not strain AD169 (MOI = 5, 24-hr infection). (D) Immunoblot showing UL145 is necessary for downregulation of HLTF (HCMV U_L_/*b*′ single gene-deletion viruses used at MOI = 5, 72-hr infection). (E) Immunoblot of stably transduced HFFF-TERTs showing UL145 is sufficient for downregulation of HLTF. (F) UL145 protein is expressed from at least 6 hr post infection (earliest time point studied in pSILAC 0- to 18-hr data, Figure 2). UL145 RNA was detected by RNA-seq from 24 hr post infection, the earliest time point studied in the RNA/protein screen (Figure 3). (G) Detection of UL145 transcript from 6 hr of infection at MOI = 1 by qRT-PCR. Error bars show SEM for technical quadruplicates.

HCMV is the largest human herpesvirus, potentially encoding hundreds of proteins (Mocarski et al., 2013, Stern-Ginossar et al., 2012). Given the large and uncertain numbers of functional proteins, identification of which viral protein targets a given cellular factor can be a challenging task. We took a systematic approach to identify the proteins targeting HLTF, initially by employing a panel of recombinant viruses deleted for one or other of a series of blocks of genes non-essential for replication *in vitro* (Fielding et al., 2014) (Table S5A). Ten block deletion viruses were screened in two parallel multiplexed proteomic analyses, with most blocks analyzed in biological duplicate. For each human protein target, a *Z* score and fold change (FC) compared with wild-type (wt) infection were calculated as described in STAR Methods. To confidently assign modulated cellular proteins to viral blocks, stringent criteria with a final *Z* score of >6 and FC >2 assigned 91 proteins, and sensitive criteria with a final *Z* score of >5 and FC >1.5 assigned 251 proteins (Figures 5A, 5B, and S6A, all predictions shown in Table S6).

The data were validated from multiple positive controls, including the 14 known targets of the US1-US11 block, such as HLA-A, -B, and -C molecules, α integrins ITGA2, 4, and 6, and butyrophilin 2A1 (BTN2A1) (Hsu et al., 2015). Nineteen known targets of the US18-US22 block were additionally confirmed, as well as our previous report that ABCC1 is targeted by UL138, a U_L_/*b*′ gene (Figures 5A, 5B, and S6B; Table S6) (Fielding et al., 2014, Weekes et al., 2013). HLTF was targeted by a single viral block, U_L_/*b*′, and this was confirmed by immunoblot (Figures 5A and 5C).

This analysis also enabled an examination of which blocks of viral genes are most important in the regulation of host factors. There was striking block-to-block variation, with three blocks, US1-US11, US18-US22, and U_L_/*b*′ each regulating >15 (stringent) or >35 (sensitive) proteins (Figure 5B). By sensitive criteria, the US12-US17 block was similarly important, regulating 59 proteins (Figure S6A). It is possible that protein *Z* scores from this block were lower due to frequent co-regulation of protein targets with the US18-US22 block (Fielding et al., 2017). Other blocks had few or no protein targets, suggesting that the proteins they encode may not be dominantly directed toward regulation of the host proteome.

To determine which individual proteins target HLTF for degradation, we generated a library of HCMV mutants with deletions of single canonical genes in U_L_/*b*′ (Table S5B). Only deletion of UL145 rescued expression of HLTF (Figure 5D). Overexpression of a C-terminally V5-tagged UL145 (UL145-V5) was sufficient to downregulate HLTF, and the expression of both proteins was rescued by MG132, which may suggest co-degradation in the proteasome (Figure 5E). UL145 was one of the viral proteins most substantially rescued by MG132, at each time point studied in the inhibitor screen (Figures S2C and S6C). Both UL145 transcript and newly synthesized protein were detected from 6 hr of infection (Figures 5F and 5G), confirming that the protein is expressed sufficiently early to regulate HLTF.

The gene encoding UL145 is located between UL144 and UL146, which exhibit high sequence variability (Dolan et al., 2004). In contrast, UL145 is well conserved (Sun et al., 2007), with our assessment of UL145 sequences from 242 genome sequences indicating identity levels of 80% and 83% at the nucleotide and amino acid sequence levels, respectively (data not shown). The presence of UL145 orthologs in Old and New World primate cytomegaloviruses indicates that this gene has existed for many millions of years (Figure S6D). Thus, although UL145 is not essential for viral replication *in vitro*, it is likely to play an important role in promoting HCMV persistence.

### HCMV UL145 Recruits the Cullin4 E3 Ligase Complex to Target HLTF to the Proteasome

**Figure 6:**
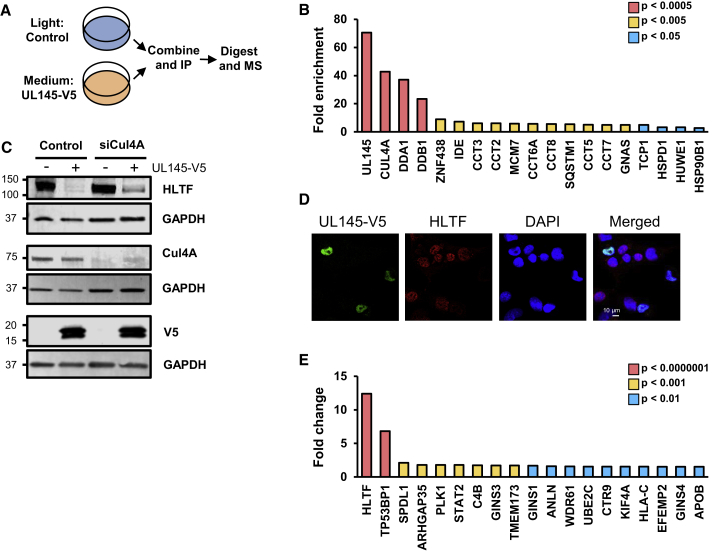
HCMV UL145 Degrades HLTF via the Cullin 4E3 Ligase Complex, and Additionally Targets TP53BP1 (A) Schematic of SILAC immunoprecipitation (IP). MS, mass spectrometry. (B) Results of SILAC immunoprecipitation. The fold enrichment of each protein is shown. p values were estimated using significance A values, then corrected for multiple hypothesis testing (Cox and Mann, 2008). Proteins enriched with p < 0.05 are shown in the graph. (C) Immunoblot showing HCMV UL145 downregulates HLTF in a CUL4A-dependent manner. 293T cells stably expressing UL145-V5 or vector control were treated with control siRNA, or siRNA against CUL4A for 48 hr. (D) Immunofluorescence demonstrated nuclear localization of UL145 (MOI = 0.1, 24-hr infection with Merlin strain recombinant with a C-terminal UL145 V5 tag). (E) UL145 targets TP53BP1 in addition to HLTF. HFFF-TERTs were infected with WTor ΔUL145 HCMV at MOI = 5 for 72 hr. Shown are proteins quantified by ≥2 peptides and rescued >1.5-fold both by ΔUL145 compared with wt, and by the U_L_/*b*′ block deletion compared with WT (Figure 5A). Values displayed are the minimum fold change and the maximum p value from the ΔUL145/wt and U_L_/*b*′/wt experiments. Benjamini-Hochberg-corrected significance A was used to estimate p values.

To identify cellular factors interacting with UL145, we performed a SILAC immunoprecipitation in HFFF-TERTs stably expressing UL145-V5 (Figure 6A). Cullin 4A (CUL4A) and adaptor molecules damage-specific DNA-binding protein 1 (DDB1) and DET1- and DDB1-associated protein 1 (DDA1) all co-precipitated with UL145 (Figure 6B). Small interfering RNA (siRNA) knockdown of CUL4A inhibited UL145-mediated HLTF downregulation, suggesting that UL145 may redirect the Cullin4 ligase complex to degrade HLTF, in a similar manner to HIV Vpr (Figure 6C). Vpr and UL145 are both small, soluble 14-kDa proteins. Vpr is known to form three α helices folded around a hydrophobic core; this structure is important for interactions with HLTF and other targets, including uracil DNA glycosylase (UNG) (Wu et al., 2016). UL145 is also predicted to form three α helices (Figure S6D), hinting that both proteins may have evolved a similar structure to degrade HLTF.

HLTF is known to localize predominantly to the nucleus, consistent with its function in DNA repair (Sheridan et al., 1995). To determine the subcellular localization of UL145, we generated a recombinant Merlin strain HCMV with a C-terminal UL145 V5-tag. As expected, infected cells exhibited very low-level expression of HLTF (Figure 6D). Both proteins exhibited a predominantly nuclear localization, although some HLTF formed perinuclear cytoplasmic speckles. Cullin4A localizes to the nucleus via a nuclear localization signal (Jackson and Xiong, 2009). The Cullin4A-mediated degradation of HLTF may therefore occur via the nuclear ubiquitin-proteasome system.

To determine whether UL145 has other cellular targets in addition to HLTF, we performed an unbiased proteomic comparison of HFFF-TERTs infected with WT or ΔUL145 virus. The double-strand break repair protein tumor protein p53-binding protein 1 (TP53BP1) was rescued both by ΔUL145 and ΔU_L_/*b*′ viruses, compared with WT infection (Figures 6E and S6E), suggesting that UL145 may have wider roles in modulating the DNA-damage response.

### HLTF Restricts HCMV Early in Infection

**Figure 7:**
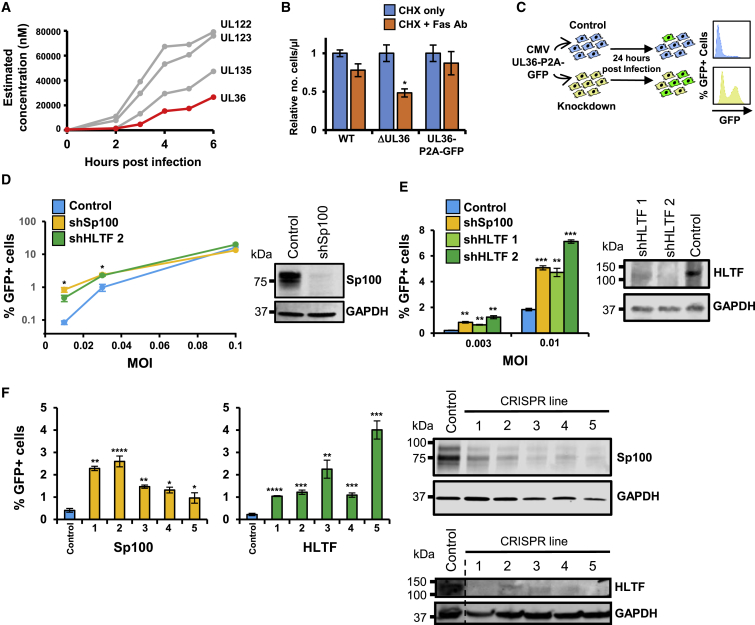
HLTF Restricts Early HCMV Infection (A) Viral proteins with the highest cellular concentration, estimated using a “proteomic ruler” approach (Wisniewski et al., 2014) with data from the 0- to 6-hr pSILAC experiment. A UL123 C-terminal GFP tag impaired viral growth. A similar problem occurred with UL135 (data not shown). The inhibitor of apoptosis UL36, which was the fourth most abundant viral protein, was therefore selected and C-terminally tagged with a self-cleaving P2A peptide followed by EGFP. (B) A C-terminal UL36-GFP tag does not impair protein UL36 function. Cells were infected with the indicated virus for 24 hr, then treated with cycloheximide (CHX) and a crosslinking Fas antibody. A viability dye was used to quantify live cells by flow cytometry. Data were normalized to the number of live cells in the absence of Fas antibody. UL36-P2A-GFP virus was comparable with wt, whereas virus lacking UL36 was significantly more sensitive to Fas-mediated apoptosis. ^∗^p < 0.05 by two-way ANOVA (n = 3). (C) Schematic of the restriction assay. HFFF-TERTs were infected at low MOI after stable knockdown for a putative restriction factor or control. (D) Validation of the restriction assay using shRNA. Representative results from one of four experiments are shown (left panel). At MOI = 0.1, a difference between shSp100 and shControl cells was no longer detectable, suggesting that the antiviral activity of Sp100 was efficiently overcome. In the same experiment, HLTF restricted infection similarly to Sp100. Values shown are mean ± SEM p values for a difference between shSp100 or shHLTF, and control cells were estimated using a two-tailed t test (n = 3). ^∗^p < 0.05 (for both Sp100 and HLTF where indicated). Immunoblot confirmed knockdown of all Sp100 isoforms (right panel) and HLTF (E). (E) HLTF restricts early HCMV infection. Application of the restriction assay at low MOI using two different shHLTF vectors suggested that HLTF restricted infection at least as potently as Sp100 (left panel). p values were estimated using a two-tailed t test (n = 3). ^∗∗^p < 0.005, ^∗∗∗^p < 0.0005. (F) Confirmation that HLTF restricts early HCMV infection using five independent polyclonal CRISPR/Cas9 Sp100 and HLTF cell lines. Each employed integrated guide RNAs (gRNAs) with different target sequences within a given gene. Control cells expressing non-targeting gRNAs were generated in a similar manner (right panels; superfluous lanes from the HLTF and corresponding GAPDH gel have been digitally eliminated as indicated by the dashed line). Infection at MOI = 0.01 identified a substantial increase in viral replication in knockdown compared with control cells (left panels). p values were estimated using a two-tailed t test (n = 3). ^∗^p < 0.05, ^∗∗^p < 0.005, ^∗∗∗^p < 0.0005, ^∗∗∗∗^p < 0.00005.

We sought to determine whether HLTF acts as a restriction factor. To identify HCMV-infected cells, we cloned enhanced GFP (EGFP) as a C-terminal fusion with the immediate-early gene UL36, with a self-cleaving P2A peptide releasing the reporter following synthesis. UL36 was chosen for this analysis since we found it to be among the most abundantly expressed viral proteins within the first 6 hr of infection, and the insertion of GFP did not impede UL36 function (Figures 7A and 7B). We adapted an assay previously deployed to examine the role of PML-NB components in HCMV restriction (Tavalai et al., 2011) (Figure 7C). The PML-NB protein Sp100 acts to restrict HCMV infection and was thus selected as a positive control. Sp100 depletion consistently enhanced HCMV UL36-GFP expression in four independent experiments (Figure 7D). This effect was highly dependent on the viral dose. The enhancement of virus infection with Sp100 knockdown was much more pronounced at lower MOIs as has been previously reported, possibly explained by efficient viral antagonism of Sp100 at higher MOI (Tavalai et al., 2011). Remarkably similar results were observed with short hairpin RNA (shRNA) knockdown of HLTF. At low MOI, knockdown of HLTF significantly increased the efficiency of virus infection in two independent HFFF-TERT lines stably transduced with different HLTF shRNA constructs (Figures 7D and 7E). The enhancement of HCMV infection was confirmed using five independently derived CRISPR/Cas9 knockdown lines for both Sp100 and HLTF (Figure 7F). HLTF thus acts to restrict significantly the efficiency with which a low MOI HCMV infection activates immediate-early gene expression, with an efficiency similar to that of the recognized HCMV restriction factor Sp100.

## Discussion

Herpesviruses achieve lifelong persistence in infected individuals by utilizing a wide range of strategies to modulate innate and adaptive immunity. These include the deployment of proteins to target host factors for degradation. For example, the HCMV US2 protein targets at least ten cell-surface molecules to the proteasome, including the NK-activating ligand CD112 and major histocompatibility complex class I (Hsu et al., 2015). The ten members of the US12 gene family act in concert to suppress the expression of cell-surface immune ligands, with many targeted for lysosomal degradation (Fielding et al., 2017). The degradation of intrinsic cellular restriction factors (e.g., Sp100, DAXX, and MORC3) is induced by virion components or viral proteins expressed early in infection and dramatically enhances the efficiency of infection (Schreiner and Wodrich, 2013, Sloan et al., 2016, Tavalai et al., 2011).

We now provide a searchable database that systematically details the synthesis and degradation of >10,000 cellular and viral proteins during the establishment of a productive HCMV infection. Our data provide a significant insight into how this virus regulates the stability of each protein including the route and rate of degradation, and predict molecules of key importance in innate antiviral immunity to HCMV. Furthermore, data from a comprehensive panel of viral mutants enable identification of the viral genes that target >250 host proteins, and have distinguished four key genetic “hubs” of regulation, including UL13-UL20, UL133-UL150 (U_L_/*b*′), US1-US11, and US27-US28. Further information could now be gained by studying individual gene-deletion mutants for each of these hubs.

HCMV orchestrates the regulation of host gene expression through a relatively long replication cycle (>72 hr) to facilitate viral replication while evading immune defenses. The calculated median protein half-life of 58.4 hr in uninfected fibroblasts suggests that degradation may be the only mechanism that can achieve sufficiently rapid change in a subset of proteins downregulated early during infection. For example, ∼18% of proteins downregulated >3-fold within 24 hr of infection were regulated primarily by mRNA levels, but 32%–54% were targeted for degradation (using high- or medium-confidence criteria, respectively). Of these degraded proteins, 87%–89% were targeted to the proteasome (as assessed by MG132), suggesting that the lysosomal route is used less commonly. Interestingly, 1%–5% of proteins were degraded and also had reduced mRNA levels, suggesting that multiple regulatory mechanisms may be employed by HCMV for effective control of certain targets. For example, GJA1 has been reported to be degraded in the proteasome (Stanton et al., 2007). The MG132 and pSILAC screens confirmed this finding, although the RNA-seq and leupeptin data suggested that GJA1 is also transcriptionally downregulated and targeted to the lysosome.

In addition to degradation, mechanisms such as intracellular sequestration play a role in downregulation of proteins from organelles such as the plasma membrane (PM). For example, we and others have reported that the NK-activating ligands poliovirus receptor, MICB, and ULBP1-2 are downregulated from the cell surface while accumulating in the ER, retained by HCMV UL141 or UL16. By 24 hr of infection, such sequestered proteins were downregulated >2-fold from the PM but were not downregulated in whole-cell lysates (Weekes et al., 2014). Overall, this trend was observed for only 1.6% of PM proteins, suggesting that the predominant mechanism HCMV employs to downregulate proteins during the early phase of infection is proteasomal degradation.

Additional insights gained from these data include the quantitation of the majority (139/170) of the current set of canonical HCMV proteins. This suggests that our approach has sufficient sensitivity to reveal non-canonical gene usage. A large number (604) of additional HCMV ORFs have been identified by ribosome profiling as potentially being translated (Stern-Ginossar et al., 2012), but it is unclear how many represent functional polypeptides. We quantified 27 of these ORFs, but only four exhibited stable expression, which was defined as requiring identification by >10 peptides across all experiments and not being rescued by MG132 or leupeptin. For example, one of this set (ORFL147C) was identified by a total of 129 peptides and was rescued <1.2-fold by the inhibitors. Of the 13 ORFs identified from the six-frame translation, all but one lacked substantial conservation among HCMV strains or between HCMV and related viruses, and, where measured, were turned over rapidly in the proteasome. The exceptional ORF is a 5′-terminal extension of ORFL147C. Overall, the data are supportive of the current definition of the canonical gene set (Davison et al., 2013), but there is a case for functional investigations of a modest number of additional ORFs, including ORFL147C.

An example of the power of our techniques and data is the identification of an innate immune function in HCMV (UL145) and its cellular target (HTLF). HLTF was initially identified as a DNA-binding protein, which specifically recognized the SV40 enhancer and HIV-1 promoter (Sheridan et al., 1995). HLTF also binds directly to the promoters of the human β-globin and plasminogen activator inhibitor-1 genes to enhance expression, and associates with transcription factors Sp1 and Sp3, which can in some cases repress promoters (Ding et al., 1999, Li et al., 2004). Although the mechanism through which HLTF is able to restrict HCMV is yet to be elucidated, based on its cellular functions it may either repress HCMV gene transcription via Sp1/Sp3 or other factors, or function as an intrinsic viral DNA sensor that triggers antiviral immunity.

Only three drugs are currently available to treat HCMV infection, and all suffer from significant side effects and the threat of the development of resistance. In the context of the increasing frequency of transplantation, innovative strategies are clearly required. The identification of a potentially inhibitable interaction between a cellular restriction factor and a viral antagonist may therefore be of major therapeutic significance. Ideally, similar interactions involving several distinct antiviral pathways might be targeted simultaneously to inhibit viral replication in a way that is refractory to resistance. This illustrates the crucial potential of our data to identify additional proteins that have roles in restricting infection by HCMV or other viruses.

## STAR★Methods

### Key Resources Table

**Table:** 

REAGENT or RESOURCE	SOURCE	IDENTIFIER
**Antibodies**		

Rabbit polyclonal anti-HLTF	Abcam	Cat#ab17984; RRID:AB_444160
Mouse monoclonal anti-HCMV IE1/2 (CH160)	Abcam	Cat#ab53495; RRID:AB_882995
Mouse monoclonal anti-HCMV early antigens (6F8.2)	Merck	Cat#MAB8131; RRID:AB_95269
Mouse monoclonal anti-GAPDH	R&D Systems	Cat#MAB5718; RRID:AB_10892505
Mouse monoclonal anti-V5 (E10/V4RR)	Thermo	Cat#MA5-15253; RRID:AB_10977225
IRDye 680RD goat anti-mouse IgG	LI-COR	Cat#925-68070; RRID:AB_2651128
IRDye 800CW goat anti-rabbit IgG	LI-COR	Cat#925-32211; RRID:AB_2651127
Anti-mouse IgG Alexa Fluor 488	Cell Signaling Technologies	Cat#4408S; RRID:AB_10694704
Anti-rabbit IgG Alexa Fluor 647	Thermo	Cat#A31573; RRID:AB_2536183
Human TruStain FcX	BioLegend	Cat#422302
Rabbit polyclonal anti-Sp100	GeneTex	Cat#GTX131570; RRID:AB_2732019

**Bacterial and Virus Strains**		

HCMV Merlin	(Stanton et al., 2010)	RCMV1111
HCMV AD169	(Fielding et al., 2014)	N/A
HCMV AD169-GFP	(Fielding et al., 2014)	RCMV288
HCMV Merlin UL36-GFP	This paper	N/A
HCMV Merlin ΔRL1-UL11	(Fielding et al., 2014)	RCMV1333
HCMV Merlin ΔRL11-UL11	(Fielding et al., 2014)	RCMV2209
HCMV Merlin ΔUL2-UL11	(Fielding et al., 2014)	RCMV1293
HCMV Merlin ΔUL13-UL20	(Fielding et al., 2014)	RCMV1294
HCMV Merlin ΔUS1-US11	(Fielding et al., 2014)	RCMV1528
HCMV Merlin ΔUS12-US17	(Fielding et al., 2014)	RCMV1297
HCMV Merlin ΔUS18-US22	(Fielding et al., 2014)	RCMV1318
HCMV Merlin ΔUS27-US28	(Fielding et al., 2014)	RCMV1299
HCMV Merlin ΔUS29-US34A	(Fielding et al., 2014)	RCMV1300
HCMV Merlin ΔUL131A	This paper	RCMV1819
HCMV Merlin ΔUL132	This paper	RCMV1821
HCMV Merlin ΔUL133	This paper	RCMV1823
HCMV Merlin ΔUL135	This paper	RCMV1825
HCMV Merlin ΔUL136	This paper	RCMV1847
HCMV Merlin ΔUL138	This paper	RCMV1849
HCMV Merlin ΔUL139	This paper	RCMV1851
HCMV Merlin ΔUL140	This paper	RCMV1812
HCMV Merlin ΔUL141	This paper	RCMV1853
HCMV Merlin ΔUL142	This paper	RCMV1835
HCMV Merlin ΔUL144	This paper	RCMV1837
HCMV Merlin ΔUL145	This paper	RCMV1814
HCMV Merlin ΔUL146	This paper	RCMV1855
HCMV Merlin ΔUL147	This paper	RCMV2035
HCMV Merlin ΔUL147A	This paper	RCMV1839
HCMV Merlin ΔUL148	This paper	RCMV1841
HCMV Merlin ΔUL148A	This paper	RCMV1843
HCMV Merlin ΔUL148B	This paper	RCMV1845
HCMV Merlin ΔUL148C	This paper	RCMV1819
HCMV Merlin ΔUL148D	This paper	RCMV1821
HCMV Merlin ΔUL150	This paper	RCMV1823
*E*. *coli*. (α-Select Silver Competent Cells)	Bioline	Cat# BIO-85026

**Chemicals**, **Peptides**, **and Recombinant Proteins**		

Tandem mass tag (TMT) 10-plex isobaric reagents	Thermo Fisher	Cat# 90110
HPLC water	VWR	Cat# 23595.328
LC-MS grade Acetonitrile	Merck	Cat# 1.00029.2500
Formic acid	Thermo Fisher	Cat# 85178
MG132	Merck	Cat#474787
Leupeptin	Merck	Cat#108975
Bafilomycin A	Alfa Aesar	Cat#J61835
10X RIPA	Cell Signaling Technologies	Cat#9806S
Complete Protease Inhibitor Cocktail	Roche	Cat# 11836153001
Fixation Buffer	BioLegend	Cat#420801
DMSO	Sigma-Aldrich	Cat#D8418
Dexamethasone	Sigma-Aldrich	Cat#D4902
L-arginine monohydrochloride	Sigma-Aldrich	Cat#A6969
L-Lysine dihydrochloride	Sigma-Aldrich	Cat#L5751
Medium Arginine	CK Isotopes	Cat#CLM-2265-H
Medium Lysine	CK Isotopes	Cat#DLM-2640
Heavy Arginine	CK Isotopes	Cat#CNLM-539-H
Heavy Lysine	CK Isotopes	Cat#CNLM-291-H
SILAC DMEM	Gibco	Cat#88364
Dialysed FBS	Gibco	Cat#24600044
Proline	Sigma-Aldrich	Cat#P5607
DAPI	Cell signaling	Cat#4083S

**Critical Commercial Assays**		

BCA Protein Assay Kit	Thermo Fisher	Cat#23227
Micro BCA Protein Assay Kit	Thermo Fisher	Cat#23235
RNeasy Plus Kit	Qiagen	Cat#74134
RNeasy Mini Kit	Qiagen	Cat#74104
Poly(A)Purist MAG kit	Thermo Fisher	Cat#AM1922
PrepX RNA-Seq Library Kit	Wafergen Biosystems	Cat#400039
GoScript Reverse Transcriptase kit	Promega	Cat#A5001
TaqMan™ Universal PCR Master Mix	Thermo Fisher	Cat#4304437

**Deposited Data**		

Unprocessed peptide files for Figures 1, 2, 3, and 5	This paper	The peptide data reported in this paper have been deposited to Mendeley Data and are available at http://dx.doi.org/10.17632/zkgmjzrcyk.1
RNA-seq metadata, processed data, FASTQ files	This paper	The accession number for the RNAseq data reported in this paper is GEO: GSE111036.
Raw Mass Spectrometry Data Files	This paper	The accession number for the RNAseq data reported in this paper is PRIDE: PXD009945.

**Experimental Models**: **Cell Lines**		

Human Fetal Foreskin Fibroblast (HFFF)	(Stanton et al., 2007)	N/A
HFFF immortalized with human telomerase (HF-TERT)	(Stanton et al., 2007)	N/A

**Oligonucleotides**		

GAPDH (Hs02786624_g1)	Thermo Fisher	Cat#4331182
HLTF (Hs00172585_m1)	Thermo Fisher	Cat#4331182
Sp100 (Hs00162109_m1)	Thermo Fisher	Cat#4331182
Oligonucleotide 1 for pHAGE-pSFFV-Control construct: GGGGACAAGTTTGTACAAAAAAGCAGGCTCCCAGGCG AGAACGTGTGCGTGGACAAGCGAGCAGCATACGAACC CAGCTTTCTTGTACAAAGTGGTCCCC	This paper	N/A
Oligonucleotide 2 for pHAGE-pSFFV-Control construct: GGGGACCACTTTGTACAAGAAAGCTGGGTTCGTATGCTG CTCGCTTGTCCACGCACACGTTCTCGCCTGGGAGCCTG CTTTTTTGTACAAACTTGTCCCC	This paper	N/A
Forward primer for pHAGE-pSFFV-UL145-V5 construct: GGGGA CAAGTTTGTACAAAAAAGCAGCTGAAGACACCGGGACCGATC	This paper	N/A
Reverse primer for pHAGE-pSFFV-UL145-V5 construct: GGGGACCACTTTGTACAAGAAAGCTGGGTTTACGTAGAATC AAGACCTAGGAGC	This paper	N/A
Forward primer for HCMV UL145 RT-qPCR: CCCATCATGCGTCGTATCAC	This paper	N/A
Reverse primer for HCMV UL145 RT-qPCR: CCGACTGATCTAGCCTACGG	This paper	N/A
Forward primer for GAPDH RT-qPCR: AGGGCTGCTTTTAACTCTGGT	This paper	N/A
Reverse primer for GAPDH RT-qPCR: CCCCACTTGATTTTGGAGGGA	This paper	N/A
Please refer to Table S7C for complete oligonucleotides including those for shRNA and CRISPR/cas9 gene disruption.		

**Recombinant DNA**		

pHRSIREN-Control_1	This paper	N/A
pHRSIREN-Control_2	This paper	N/A
pHRSIREN-Sp100_1	This paper	N/A
pHRSIREN-HLTF_1	This paper	N/A
pHRSIREN-HLTF_2	This paper	N/A
pKLV-U6gRNA(BbsI)-PGKpuro2ABFP	Addgene	#50946
pHRSIN-P_SFFV_-Cas9-P_PGK_-Hygro	This paper	N/A
pHAGE-pSFFV	This paper	N/A
pHAGE-pSFFV-Control	This paper	N/A
pHAGE-pSFFV-UL145-V5	This paper	N/A

**Software and Algorithms**		

“MassPike”, a Sequest-based software pipeline for quantitative proteomics.	Professor Steven Gygi’s lab, Harvard Medical School, Boston, USA.	N/A
XLStat	Addinsoft	https://www.xlstat.com/en/
DAVID software	Huang da et al., 2009	https://david.ncifcrf.gov/
Cluster 3.0	Stanford University University of Tokyo	http://bonsai.hgc.jp/∼mdehoon/software/cluster/software.htm
Java Treeview	SourceForge.net	http://jtreeview.sourceforge.net/
Image Studio Lite	LI-COR	Ver. 5.2 https://www.licor.com/bio/products/software/image_studio_lite/
Clustal Omega	EMBL-EBI	https://www.ebi.ac.uk/Tools/msa/clustalo/
FlowJo	FlowJo	Ver. 10 https://www.flowjo.com/solutions/flowjo

**Other**		

Orbitrap Fusion Mass Spectrometer	ThermoFisher Scientific	Cat# IQLAAEGAAP FADBMBCX
Orbitrap Fusion Lumos Mass Spectrometer	ThermoFisher Scientific	Cat# IQLAAEGAAP FADBMBHQ
Apollo 324	Wafergen Biosystems	N/A
Agilent Bioanalyzer 2100	Agilent Technologies	Cat#G2939BA

### Contact for Reagent and Resource Sharing

Further information and requests for resources and reagents should be directed to and will be fulfilled by the Lead Contact, Michael Weekes (mpw1001@cam.ac.uk).

### Experimental Model and Subject Details

#### Cells and Cell Culture

Primary human fetal foreskin fibroblast cells (HFFFs, male) and HFFFs immortalised with human telomerase (HFFF-TERTs) were grown in Dulbecco’s modified Eagle’s medium (DMEM) supplemented with foetal bovine serum (FBC: 10% v/v), and penicillin/streptomycin at 37°C in 5% CO_2_. HFFFs and HFFF-TERTs have been tested at regular intervals since isolation to confirm both that the HLA & MICA genotypes and the morphology and antibiotic resistances are consistent with the original cells. In addition, the HCMV Merlin strain used is only permissive in human fibroblasts (dermal or foreskin), further limiting the chances that the cells have been contaminated with another cell type.

For pulsed SILAC analysis, cells were grown for seven divisions in DMEM for SILAC, which was supplied without light arginine or lysine. This medium was supplemented with 10% dialysed FBS, penicillin/streptomycin, 84 mg/ml light arginine, 280 mg/l L-proline and either 50 mg/ml medium lysine (Lys 4) or 146 mg/ml heavy lysine (Lys 8). For SILAC immunoprecipitations, cells were grown identically but the medium was supplemented with 10% dialysed FBS, penicillin/streptomycin, 280 mg/l L-proline and either light (Arg 0, Lys 0) or medium (Arg 6, Lys 4) amino acids at 50 mg/l. Incorporation of heavy label was >98% for both arginine and lysine-containing peptides.

#### Viruses

We used virus (RCMV1111) derived by transfection of a BAC clone of HCMV strain Merlin, the genome of which is designated the reference HCMV sequence by the National Center for Biotechnology Information and was sequenced after 3 passages *in vitro* (Dolan et al., 2004) (Stanton et al., 2010). RCMV1111 contains point mutations in two genes (RL13 and UL128) that enhance replication in fibroblasts (Stanton et al., 2010). The 10 block HCMV deletion mutants were generated on a strain Merlin background (wt1), or wt1 that lacked UL16 and UL18 (wt2) by transfection of recombinant BACs (Stanton et al., 2010) as described in (Fielding et al., 2014). The wt2 background was originally employed to facilitate detection of NK evasion functions (deletion and backbone details shown in Table S5A) (Fielding et al., 2014). HCMV strain AD169 varUK/BK000394 was used for the U_L_/*b’* mutant, as a deletion in this region (plus additional defects) has been acquired during passage in culture. Single gene deletion mutants of all the canonical genes in the U_L_/*b’* region were generated by recombineering the strain Merlin BAC as described previously (Stanton et al., 2010). Whole-genome consensus sequences of passage 1 of each RCMV were derived using the Illumina platform as described previously (Fielding et al., 2014), and deposited in GenBank. HCMV expressing rGFP from a P2A self-cleaving peptide following the UL36 ORF, and UL145-V5 recombinants were generated as described in (Fielding et al., 2014). For part of the inhibitor screen, viruses were irradiated with a dose of 3500 Gy using a Gammacell 1000 Elite (Nordion International), and inactivation was verified by the absence of immunofluorescence for IE1 compared to control (data not shown) (Weekes et al., 2014).

Virus stocks were prepared from HFFF-TERTs as described previously (Stanton et al., 2007). Tissue culture supernatants were kept when a 100% cytopathic effect was observed, and were centrifuged to remove cell debris. Cell-free virus was pelleted from supernatant by centrifugation at 22,000 × g for 2 h and then resuspended in fresh DMEM. Residual debris was removed from the resulting virus stocks by centrifugation at 16,000 x g for 1 min.

### Method Details

#### Virus Infections and Inhibitors for Proteomic Experiments

1 x 10^6^ HFFFs (RNA/protein screen, block deletion mutant screen) or HFFF-TERTs (inhibitor, pSILAC, wt vs ΔUL145 virus screens) were plated in a 25cm^2^ flask. We found that primary HFFFs were limited to a total of ∼25 passages and exhibited diminished rates of growth as passage number increased. HFFF-TERTs were therefore used in the pSILAC screen, due the need for seven cell divisions prior to infection, and in the inhibitor screen to ensure comparability of results. Cells were infected at multiplicity of infection 5 or 10 with HCMV strain Merlin as previously described (Weekes et al., 2014). Briefly, the requisite volume of viral stock was added to 1 ml DMEM and mixed gently prior to being applied to cells. Mock infections were performed identically but with additional DMEM instead of viral stock. Time 0 in any experiment was considered to be the initial point of infection with virus. In each experiment, cells were incubated with virus for 2 h prior to a change in medium with the exception of the 6 h pSILAC experiment, where cells were incubated with virus for 1.5 h. Inhibitors added at the indicated times were: 10 μM MG132 (Merck) or 200 μM Leupeptin (Merck).

#### Pulsed SILAC Analysis

For pulsed SILAC, cells were grown for seven divisions in medium-labelled SILAC DMEM as detailed above. Seeding at 1 x 10^6^ per T25 gave a confluent culture, resulting in the arrest of cell division by contact inhibition. This ensured that protein turnover was the sole determinant of labeling kinetics, as opposed to dilution of cellular proteins by cell division. At time 0, media was changed to heavy-labelled SILAC DMEM containing HCMV or equivalent virus-free medium for mock infection. Without frequent media changes, substantial recycling of medium-labelled amino acids occurred (data not shown). We therefore (a) increased the concentration of heavy lysine to that of standard DMEM, namely 146 mg/l. (b) changed media every 45 mins for the whole course of the experiment (18 h pSILAC experiment) or every 30 mins for the whole experiment (6 h pSILAC experiment). Cells were harvested at time points detailed in Figure 2A. For the 3 h time point in Experiment 2, HCMV-infected and not mock-infected cells were harvested, as only 11 TMT labelling reagents were available.

#### Whole Cell Lysate Protein Digestion

Cells were washed twice with PBS, and 250 μl lysis buffer added (6M Guanidine/50 mM HEPES pH 8.5). Cell lifters (Corning) were used to scrape cells in lysis buffer, which was removed to an eppendorf tube, vortexed extensively then sonicated. Cell debris was removed by centrifuging at 21,000 g for 10 min twice. Although this method prohibited cell counting immediately prior to lysis, it avoided the need for cellular detachment. Half of each sample was kept for subsequent analysis by immunoblot where required. For the other half, dithiothreitol (DTT) was added to a final concentration of 5 mM and samples were incubated for 20 mins. Cysteines were alkylated with 14 mM iodoacetamide and incubated 20 min at room temperature in the dark. Excess iodoacetamide was quenched with DTT for 15 mins. Samples were diluted with 200 mM HEPES pH 8.5 to 1.5 M Guanidine followed by digestion at room temperature for 3 h with LysC protease at a 1:100 protease-to-protein ratio. Samples were further diluted with 200 mM HEPES pH 8.5 to 0.5 M Guanidine. Trypsin was then added at a 1:100 protease-to-protein ratio followed by overnight incubation at 37°C. The reaction was quenched with 5% formic acid, then centrifuged at 21,000 g for 10 min to remove undigested protein. Peptides were subjected to C18 solid-phase extraction (SPE, Sep-Pak, Waters) and vacuum-centrifuged to near-dryness.

#### Peptide Labeling with Tandem Mass Tags

In preparation for TMT labeling, desalted peptides were dissolved in 200 mM HEPES pH 8.5. Peptide concentration was measured by microBCA (Pierce), and 25 μg of peptide labeled with TMT reagent. TMT reagents (0.8 mg) were dissolved in 43 μl anhydrous acetonitrile and 3 μl added to peptide at a final acetonitrile concentration of 30% (v/v). Sample labelling was as indicated in Table S7A. Following incubation at room temperature for 1 h, the reaction was quenched with hydroxylamine to a final concentration of 0.3% (v/v). TMT-labeled samples were combined at a 1:1:1:1:1:1:1:1:1:1:1 ratio. The sample was vacuum-centrifuged to near dryness and subjected to C18 SPE (Sep-Pak, Waters). An unfractionated singleshot was analysed initially to ensure similar peptide loading across each TMT channel, thus avoiding the need for excessive electronic normalization. As all normalisation factors were >0.5 and <2, data for each singleshot experiment was analysed with data for the corresponding fractions to increase the overall number of peptides quantified. Normalisation is discussed in ‘Data Analysis’, and high pH reversed-phase (HpRP) fractionation is discussed below.

#### Offline HpRP Fractionation

TMT-labelled tryptic peptides were subjected to HpRP fractionation using an Ultimate 3000 RSLC UHPLC system (Thermo Fisher Scientific) equipped with a 2.1 mm internal diameter (ID) x 25 cm long, 1.7 μm particle Kinetix Evo C18 column (Phenomenex). Mobile phase consisted of A: 3% acetonitrile (MeCN), B: MeCN and C: 200 mM ammonium formate pH 10. Isocratic conditions were 90% A/10% C, and C was maintained at 10% throughout the gradient elution. Separations were conducted at 45°C. Samples were loaded at 200 μl/minute for 5 minutes. The flow rate was then increased to 400 μl/minute over 5 minutes, after which the gradient elution proceed as follows: 0-19% B over 10 minutes, 19-34% B over 14.25 minutes, 34-50% B over 8.75 minutes, followed by a 10 minutes wash at 90% B. UV absorbance was monitored at 280 nm and 15 s fractions were collected into 96 well microplates using the integrated fraction collector. Fractions were recombined orthogonally in a checkerboard fashion, combining alternate wells from each column of the plate into a single fraction, and commencing combination of adjacent fractions in alternating rows. Wells were excluded prior to the start or after the cessation of elution of peptide-rich fractions, as identified from the UV trace. This yielded two sets of 12 combined fractions, A and B, which were dried in a vacuum centrifuge and resuspended in 10 μl MS solvent (4% MeCN/5% formic acid) prior to LC-MS3. 12 set ‘A’ fractions were used for MS analysis of all experiments. For the 18 h pSILAC (pSILAC_18) experiment, an additional 6 set ‘B’ fractions were used, as 6/12 original fractions were suboptimally analysed on the Orbitrap Lumos. For the 12 h inhibitor experiment (Deg_12), a single fraction failed to run optimally and a further set ‘B’ fraction was analysed. Both set ‘A’ and set ‘B’ runs were included in the final analysis in each case (Table S7B).

#### LC-MS3 for TMT and TMT/SILAC Experiments

Mass spectrometry data was acquired using an Orbitrap Lumos for all experiments apart from 6 fractions from the pSILAC_18 experiment, where an Orbitrap Fusion mass spectrometer was used instead (Thermo Fisher Scientific, San Jose, CA). In both cases, an Ultimate 3000 RSLC nano UHPLC equipped with a 300 μm ID x 5 mm Acclaim PepMap μ-Precolumn (Thermo Fisher Scientific) and a 75 μm ID x 50 cm 2.1 μm particle Acclaim PepMap RSLC analytical column was used.

##### For Orbitrap Lumos Experiments

Loading solvent was 0.1% FA, analytical solvent A: 0.1% FA and B: 80% MeCN + 0.1% FA. All separations were carried out at 55°C. Samples were loaded at 5 μL/minute for 5 minutes in loading solvent before beginning the analytical gradient. The following gradient was used: 3-7% B over 3 minutes, 7-37% B over 173 minutes, followed by a 4 minute wash at 95% B and equilibration at 3% B for 15 minutes. Each analysis used a MultiNotch MS3-based TMT method (McAlister et al., 2014). The following settings were used: MS1: 380-1500 Th, 120,000 Resolution, 2x10^5^ automatic gain control (AGC) target, 50 ms maximum injection time. MS2: Quadrupole isolation at an isolation width of m/z 0.7, CID fragmentation (normalised collision energy (NCE) 35) with ion trap scanning in turbo mode from m/z 120, 1.5x10^4^ AGC target, 120 ms maximum injection time. MS3: In Synchronous Precursor Selection mode the top 6 MS2 ions were selected for HCD fragmentation (NCE 65) and scanned in the Orbitrap at 60,000 resolution with an AGC target of 1x10^5^ and a maximum accumulation time of 150 ms. Ions were not accumulated for all parallelisable time. The entire MS/MS/MS cycle had a target time of 3 s. Dynamic exclusion was set to +/- 10 ppm for 70 s. MS2 fragmentation was trigged on precursors 5x10^3^ counts and above.

##### For Orbitrap Fusion Experiments

Loading solvent was 0.1% TFA, analytical solvent A: 0.1% FA and B: MeCN + 0.1% FA. All separations were carried out at 55°C. Samples were loaded at 10 μl/minute for 5 minutes in loading solvent before beginning the analytical gradient. The following gradient was used: 3-5.6% B over 4 minutes, 5.6-32% B over 162 minutes, followed by a 5 minute wash at 80% B and a 5 minute wash at 90% B and equilibration at 3% B for 5 minutes. Each analysis used a MultiNotch MS3-based TMT method (McAlister et al., 2014). The following settings were used: MS1: 400-1400 Th, Quadrupole isolation, 120,000 Resolution, 2x10^5^ AGC target, 50 ms maximum injection time, ions injected for all parallisable time. MS2: Quadrupole isolation at an isolation width of m/z 0.7, CID fragmentation (NCE 30) with ion trap scanning out in rapid mode from m/z 120, 1x10^4^ AGC target, 70 ms maximum injection time, ions accumulated for all parallisable time in centroid mode. MS3: in Synchronous Precursor Selection mode the top 10 MS2 ions were selected for HCD fragmentation (NCE 65) and scanned in the Orbitrap at 50,000 resolution with an AGC target of 5x10^4^ and a maximum accumulation time of 150 ms, ions were not accumulated for all parallelisable time. The entire MS/MS/MS cycle had a target time of 3 s. Dynamic exclusion was set to +/- 10 ppm for 90 s. MS2 fragmentation was trigged on precursors 5x10^3^ counts and above.

#### Immunoprecipitation and Protein Digestion

Cells were harvested in lysis buffer (50 mM Tris pH 7.5, 300 mM NaCl, 0.5% (v/v) NP40, 1 mM DTT and Roche protease inhibitor cocktail), tumbled for 15 minutes at 4°C and then centrifuged at 16,100 g for 20 minutes at 4°C. Lysates were then clarified by filtration through a 0.7 μm filter and incubated for 3 h with immobilised mouse monoclonal anti-V5 agarose resin. Samples were washed multiple times with lysis buffer, followed by multiple PBS pH 7.4 washes. Subsequently, proteins bound to the anti-V5 resin were eluted twice by adding 200 μl of 250 μg/ml V5 peptide (Alpha Diagnostic International) in PBS at 37°C for 30 minutes with agitation. Finally, proteins were precipitated with 20% TCA, washed once with 10% TCA, washed three times with cold acetone and dried to completion using a centrifugal evaporator. Samples were re-suspended in protein loading dye, electrophoresed approximately 2 cm into a precast SDS-Polyacrylamide gel and stained with SimplyBlue Safe Stain (Novex). The lane was excised, and the proteins digested in-gel for mass spectrometry on the Orbitrap Lumos.

#### LC-MS/MS for Immunoprecipitation Experiments

Loading solvent was 3% MeCN, 0.1% FA, analytical solvent A: 0.1% FA and B: MeCN + 0.1% FA. All separations were carried out at 55°C. Samples were loaded at 5 μl/minute for 5 minutes in loading solvent before beginning the analytical gradient. The following gradient was used: 3-40% B over 29 minutes followed by a 3 minute wash at 95% B and equilibration at 3% B for 10 minutes. The following settings were used: MS1: 300-1500 Th, 120,000 resolution, 4x10^5^ AGC target, 50 ms maximum injection time. MS2: Quadrupole isolation at an isolation width of m/z 1.6, HCD fragmentation (NCE 35) with fragment ions scanning in the Orbitrap from m/z 110, 5x10^4^ AGC target, 60 ms maximum injection time, ions accumulated for all parallelisable time. Dynamic exclusion was set to +/- 10 ppm for 60 s. MS2 fragmentation was trigged on precursors 5x10^4^ counts and above.

#### RNAseq Analysis

RNAseq analysis was performed in biological triplicate at three time points of infection: 0h (mock), 24 h and 72 h. For each sample, RNA was extracted from a 75 cm^2^ flask of HFFFs infected at moi 10 or mock-infected using an RNeasy Plus kit (Qiagen). Infections and harvests were performed simultaneously with protein samples for experiment WCL2 (Weekes et al., 2014). Poly(A) RNA was enriched using a Poly(A)Purist MAG kit (Thermo). 250 ng of poly(A) RNA from each sample was used to prepare a cDNA library using a PrepX RNA-Seq Library Kit (Wafergen biosystems) on an Apollo 324 (WaferGen biosystems), according to the manufacturer's protocol. The following barcode sequences were used: Mock1 (ATCACGAT); Mock2 (CGATGTAT); Mock3 (TTAGGCAT); 24h_1 (TGACCAAT); 24h_2 (ACAGTGAT); 24h_3 (GCCAATAT); 72h_1 (CAGATCAT); 72h_2 (ACTTGAAT); 72h_3 (GATCAGAT). The resulting libraries were quantified using an Agilent Bioanalyser 2100 (Agilent Technologies, Santa Clara, CA), then were pooled for sequencing on a single lane of Illumina HiSeq2500 (1 × 50 bp reads).

#### Plasmid Construction

For exogenous gene expression, a V5-tagged UL145 construct was amplified from an adenoviral template, which expressed UL145-V5 under the control of a CMV promoter. Primers were designed to recognise the 3’ end of the CMV promoter (forward) and the V5 tag (reverse) (Key Resources Table). A control construct was prepared by annealing two oligonucleotides. Both primers and oligonucleotides had flanking Gateway attB sequences (Key Resources Table). PCR employed the PfuUltra II Fusion HS DNA Polymerase (Agilent). Constructs were subsequently cloned into lentiviral destination vector pHAGE-pSFFV using the Gateway system (Thermo Scientific). pHAGE-pSFFV has a spleen focus-forming virus (SFFV) promoter replacing the CMV promoter in pHAGE-pCMV to prevent promoter inactivation during HCMV infection. For shRNA, two partially complementary oligonucleotides (Table S7C) were annealed. The resulting product was ligated into the pHR-SIREN vector (gift from Prof. Paul Lehner, University of Cambridge) as a BamHI–EcoRI fragment using T4 ligase (Thermo Scientific). All constructed plasmids were transformed into Alpha-Select Silver Efficiency Competent E. coli cells (Bioline) at 42°C for 1 min and selected on antibiotic-containing LB agar plates.

#### Stable Cell Line Production

Lentiviral particles were generated through transfection of HEK293T cells with the lentiviral transfer vector plus four helper plasmids (VSVG, TAT1B, MGPM2, CMV-Rev1B), using TransIT-293 transfection reagent (Mirus) according to the manufacturer's recommendations. Viral supernatant was typically harvested 48 h after transfection, cell debris was removed with a 0.22 μm filter, and target cells were transduced for 48 h then subjected to antibiotic selection for two weeks.

#### CRISPR/Cas9-Mediated Gene Disruption

HFFF-TERT cells stably expressing pHRSIN-P_SFFV_-Cas9-P_PGK_-Hygro (gift from Professor Paul Lehner, University of Cambridge) were transduced with lentivirus employing the pKLV-U6gRNA(BbsI)-PGKpuro2ABFP plasmid (Addgene Plasmid #50946), that constitutively expressed a given gRNA (Table S7C). Confirmation of protein level reduction in low passage polyclonal populations of cells expressing both Cas9 and the gRNA of choice was then achieved by immunoblot. Polyclonal selected cell populations were used in this study.

#### siRNA Knockdown

24 h prior to transfection, 3x10^5^ 293Ts constitutively expressing UL145-V5 or control were plated in 6 well plates. Cells were transfected with a pool of CUL4A siRNAs (L-012610-00, Dharmafect) or a pool of non-targeting siRNAs (D-001810-10, Dharmafect) with DHARMAfect 1 Transfection Reagent (T-2001, Dharmafect) giving a final siRNA concentration of 25 nM. Cellular lysates were harvested 48 h post transfection for immunoblot.

#### Immunoblotting

HFFF-TERTs were used for all experiments apart from Figure 6C, where 293T cells were used. For most immunoblots, cells were lysed with RIPA buffer (Cell Signaling) containing Complete Protease Inhibitor Cocktail (Roche) and then lysates were sonicated. For cells infected by single gene deletion viruses, 6 M Guanidine whole cell lysates were precipitated using a ProteoExtract protein precipitation kit (Calbiochem) and re-dissolved in 2% SDS/Tris 200 mM pH 8.5 with sonication. Protein concentration was measured by BCA (Pierce). Lysates were reduced with 6X Protein Loading Dye (Tris 375 mM pH 6.8, 12% SDS, 30% glycerol, 0.6 M DTT, 0.06% bromophenol blue) for 5 min at 95°C. 50 μg of protein for each sample was separated by PAGE using 4-15% TGX Precast Protein Gels (Bio-rad), then transferred to PVDF membranes using Trans-Blot Systems (Bio-rad). The following primary antibodies were used: anti-HLTF (ab17984, Abcam), anti-HCMV IE1/2 (ab53495, Abcam), anti-GAPDH (MAB5718, R&D Systems), anti-V5 (MA5-15253, Thermo), anti-Sp100 (GTX131570, GeneTex). Secondary antibodies were IRDye 680RD goat anti-mouse (925-68070, LI-COR) and IRDye 800CW goat anti-rabbit (925-32211, LI-COR). Fluorescent signals were detected using a LI-COR Odyssey, and images were processed using Image Studio Lite (LI-COR).

#### Restriction Assay and Flow Cytometry

24 h prior to infection, 1.5x10^5^ HFFF-TERTs stably expressing shRNA constructs targeted against Sp100, HLTF or control were plated in 24 well plates. Cells were infected with HCMV UL36-GFP at a range of low moi (0.003 – 0.3). The requisite volume of viral stock was added to 150 μl DMEM, and mixed gently prior to being applied to cells. Cells were incubated with virus for 2 h prior to replacing the medium. 24 h after infection, cells were harvested and fixed in 4% paraformaldehyde. 30,000 events were acquired with a FACSCalibur flow cytometer and analysed with FlowJo vX software. A similar approach was performed for polyclonal selected CRISPR cell populations.

#### Immunofluorescence Microscopy

HFFF-TERTs were infected on coverslips with a recombinant Merlin strain with a C-terminal UL145 V5 tag, at moi 0.1 for 24 h. Cells were then cross-linked with fixation buffer (Biolegend), permeabilised with ice-cold methanol, and blocked with Human TruStain FcX (Biolegend). Two primary antibodies were used: rabbit anti-HLTF (ab17984, Abcam) and mouse anti-V5 (MA5-15253, Thermo). Secondary antibodies were anti-mouse Alexa Fluor 488 (4408S, Cell Signaling) and anti-rabbit Alexa Fluor 647 (A31573, Thermo). Cell nuclei were stained with DAPI (Cell Signaling). Fluorescence were observed using a confocal microscope (Zeiss LSM 710).

#### RT-qPCR

Total RNA from mock- or HCMV-infected HFFF-TERTs was extracted using an RNeasy Mini Kit (Qiagen). cDNA was synthesized using GoScript Reverse Transcriptase (Promega), followed by RT-qPCR using Fast SYBR Green Master Mix (Applied Biosystems) and 7500 Fast & 7500 Real-Time PCR Systems (Applied Biosystems). Primers targeting HCMV UL145 or GAPDH (as an internal control) are shown in the Key Resources Table. The PCR program started with activation at 95°C for 2 min, followed by 40 cycles of denaturation at 95°C for 5 s and annealing/extension at 60°C for 30 s. Melting curve analyses were performed to verify the amplification specificity. All mock-infected samples exhibited non-singular melting curves, indicating non-specific amplification; values for these samples were set to zero.

### Quantification and Statistical Analysis

#### Data Analysis

Mass spectra were processed using a Sequest-based software pipeline for quantitative proteomics, “MassPike”, through a collaborative arrangement with Professor Steven Gygi’s laboratory at Harvard Medical School. MS spectra were converted to mzXML using an extractor built upon Thermo Fisher’s RAW File Reader library (version 4.0.26). In this extractor, the standard mzxml format has been augmented with additional custom fields that are specific to ion trap and Orbitrap mass spectrometry and essential for TMT quantitation. These additional fields include ion injection times for each scan, Fourier Transform-derived baseline and noise values calculated for every Orbitrap scan, isolation widths for each scan type, scan event numbers, and elapsed scan times. This software is a component of the MassPike software platform and is licensed by Harvard Medical School.

A combined database was constructed from (a) the human Uniprot database (26^th^ January, 2017), (b) the HCMV strain Merlin Uniprot database, (c) all additional non-canonical human cytomegalovirus ORFs described by Stern-Ginossar et al. (Stern-Ginossar et al., 2012), (d) a six-frame translation of HCMV strain Merlin filtered to include all potential ORFs of ≥8 amino acids (delimited by stop-stop rather than requiring ATG-stop) and (e) common contaminants such as porcine trypsin and endoproteinase LysC. ORFs from the six-frame translation (6FT-ORFs) were named as follows: 6FT_Frame_ORFnumber_length, where Frame is numbered 1-6, and length is the length in amino acids. The combined database was concatenated with a reverse database composed of all protein sequences in reversed order. Searches were performed using a 20 ppm precursor ion tolerance. Fragment ion tolerance was set to 1.0 Th. TMT tags on lysine residues and peptide N termini (229.162932 Da) and carbamidomethylation of cysteine residues (57.02146 Da) were set as static modifications, while oxidation of methionine residues (15.99492 Da) was set as a variable modification. For SILAC analysis, the following variable modifications were used: heavy lysine (8.01420 Da), heavy arginine (10.00827 Da), medium lysine (4.02511 Da), medium arginine (6.02013 Da). SILAC-only searches were performed in the same manner, omitting the TMT static modification.

To control the fraction of erroneous protein identifications, a target-decoy strategy was employed (Huttlin et al., 2010). Peptide spectral matches (PSMs) were filtered to an initial peptide-level false discovery rate (FDR) of 1% with subsequent filtering to attain a final protein-level FDR of 1%. PSM filtering was performed using a linear discriminant analysis, as described previously (Huttlin et al., 2010). This distinguishes correct from incorrect peptide IDs in a manner analogous to the widely used Percolator algorithm (https://noble.gs.washington.edu/proj/percolator/), though employing a distinct machine learning algorithm. The following parameters were considered: XCorr, ΔCn, missed cleavages, peptide length, charge state, and precursor mass accuracy.

Protein assembly was guided by principles of parsimony to produce the smallest set of proteins necessary to account for all observed peptides (algorithm described in Huttlin et al., 2010). Where all PSMs from a given HCMV protein could be explained either by a canonical gene or non-canonical ORF, the canonical gene was picked in preference.

In eleven cases, PSMs assigned to a non-canonical or 6FT-ORF were a mixture of peptides from the canonical protein and the ORF. This most commonly occurred where the ORF was a 5’-terminal extension of the canonical protein (thus meaning that the smallest set of proteins necessary to account for all observed peptides included the ORFs alone). In these cases, the peptides corresponding to the canonical protein were separated from those unique to the ORF, generating two separate entries. In a single case, PSM were assigned to the 6FT-ORF 6FT_6_ORF1202_676aa, which is a 5’-terminal extension of the non-canonical ORF ORFL147C. The principles described above were used to separate these two ORFs.

Proteins were quantified by summing TMT reporter ion counts across all matching peptide-spectral matches using ”MassPike”, as described previously (McAlister et al., 2014). Briefly, a 0.003 Th window around the theoretical m/z of each reporter ion (126, 127n, 127c, 128n, 128c, 129n, 129c, 130n, 130c, 131n, 131c) was scanned for ions, and the maximum intensity nearest to the theoretical m/z was used. The primary determinant of quantitation quality is the number of TMT reporter ions detected in each MS3 spectrum, which is directly proportional to the signal-to-noise (S:N) ratio observed for each ion. Conservatively, every individual peptide used for quantitation was required to contribute sufficient TMT reporter ions (minimum of ∼500 per spectrum) so that each on its own could be expected to provide a representative picture of relative protein abundance (McAlister et al., 2014). An isolation specificity filter with a cutoff of 50% was additionally employed to minimise peptide co-isolation (McAlister et al., 2014). Peptide-spectral matches with poor quality MS3 spectra (more than 9 TMT channels missing and/or a combined S:N ratio of less than 100 across all TMT reporter ions) or no MS3 spectra at all were excluded from quantitation. Peptides meeting the stated criteria for reliable quantitation were then summed by parent protein, in effect weighting the contributions of individual peptides to the total protein signal based on their individual TMT reporter ion yields. Protein quantitation values were exported for further analysis in Excel.

For protein quantitation, reverse and contaminant proteins were removed, then each reporter ion channel was summed across all quantified proteins and normalised assuming equal protein loading across all channels. For further analysis and display in Figures, fractional TMT signals were used (i.e. reporting the fraction of maximal signal observed for each protein in each TMT channel, rather than the absolute normalized signal intensity). This effectively corrected for differences in the numbers of peptides observed per protein.

For pulsed SILAC experiments, after protein assembly, medium-labelled peptides (measuring protein degradation), and heavy-labelled peptides (measuring protein synthesis) were extracted then re-assembled into medium- and heavy-labelled proteins using an in-house script written in Python (version 2.7). Protein normalisation across reporter ion channels again assumed equal protein loading (i.e. for each TMT channel, the summed protein S:N including all medium- and heavy-labelled proteins was the same). For each protein, values were further normalised to the time 0 sample for display in Figures. For all TMT or pSILAC experiments, normalised S:N values are presented in Table S1 (‘Data’ worksheet). For SILAC immunoprecipitations, normalisation assumed equal protein loading across all samples.

Although peptides were assigned appropriately to HLA-A alleles, it was not possible confidently to assign peptides to only two HLA-B or HLA-C alleles. For the 5 HLA-B or HLA-C alleles that had the greatest summed number of peptides across all experiments, signal:noise values were further summed to give a single combined result for HLA-B or HLA-C.

Hierarchical centroid clustering based on uncentered Pearson correlation, and k-means clustering were performed using Cluster 3.0 (Stanford University) and visualised using Java Treeview (http://jtreeview.sourceforge.net) unless otherwise noted. Multiple sequence alignment was performed using Clustal Omega (http://www.ebi.ac.uk/Tools/msa/clustalo/) provided by EMBL-EBI.

#### Comparative Data Analysis Using Proteome Discoverer

To compare data generated using “MassPike” with another platform, we re-analysed raw MS files for the 12, 18 and 24 h inhibitor experiments using Proteome Discoverer 2.2 (Thermo Fisher Scientific) (Figure S7). Data were searched in Sequest against an identical combined database to that described in the “Data analysis” section. Searches were performed using a 20 ppm precursor ion tolerance and fragment mass tolerance of 0.5 Da. TMT tags on lysine residues and peptide N termini and carbamidomethylation of cysteine residues were set as static modifications, and oxidation of methionine residues was set as a variable modification. Percolator (https://noble.gs.washington.edu/proj/percolator/) was used to control the fraction of erroneous protein identifications, with a peptide false discovery rate of 1% for ‘high’ confidence PSMs and 5% for ‘medium’ confidence PSMs. Proteins were subsequently filtered to attain a final protein-level FDR of 1% (‘strict’ criteria) or 5% (‘relaxed’ criteria). Protein assembly was guided by principles of parsimony. MS3 spectra were used for reporter ion based quantitation with a most intense centroid tolerance of 20 ppm. An average reporter S:N value of 10 was used for quantitation and an isolation specificity filter with a cutoff of 50% was employed to minimize peptide co-isolation. Peptides meeting these criteria were summed by parent protein, and quantitation values were exported for further analysis in Excel. For protein quantitation, reverse and contaminant proteins were removed, and then each reporter ion channel was summed across all quantified proteins and normalised assuming equal protein loading across all channels.

#### Histone Proteomic Ruler

The cellular concentrations of viral proteins in whole cell lysates from the 6 h pSILAC experiment (Figure 7A) were calculated using a ‘proteomic ruler’ approach implemented in the Perseus plugin (http://www.coxdocs.org/doku.php?id=perseus:user:plugins:proteomicruler:estimatecopynumbers) (Wisniewski et al., 2014). This used the mass spectrometry signal of histones from the 6 h HCMV-infected sample to scale other proteins of unknown concentration from the same sample. Briefly, intensity values that had been normalised assuming equal protein loading across all samples were imported into Perseus. Molecular weights of all human and canonical HCMV proteins were obtained from Uniprot. Predicted masses of non-canonical HCMV ORFs and six-frame translations were obtained using an online molecular weight calculator (https://www.bioinformatics.org/sms/prot_mw.html). Scaling was using the histone proteomic ruler, assuming a ploidy of 2.

#### RNAseq Data Analysis

RNA samples were collected in biological triplicate. Reads were aligned to the Human genome (hg19) and HCMV Merlin strain genome (NC_006273.2) using the aligner STAR version 2.5.2b (http://code.google.com/p/rna-star/). Read counts for each gene/transcript were determined using HTSeq version 0.6.1 (https://pypi.org/project/HTSeq/) with the following optional parameters: --m union,-r pos,-i transcript_id,-a 10,--stranded=no. For human genes, normalisation assumed equal human reads per run; HCMV reads were not included in this normalisation since by 72 h HCMV accounted for a significant proportion of all reads (0h: <0.01%, 24 h: 4.7-5.8%; 72 h: 41.8-42.3% HCMV reads). For HCMV, reads for each barcode were normalised assuming equal total reads (human plus HCMV) per sequencing run. Reads per Kilobase per Million (RPKM) values were calculated in Excel and further normalised to 1. Mean and standard error of the mean (SEM) values are shown in Table S1 and Figures 3, 5, S1–S3, and S5. Although data for HCMV reads is included in Table S1, this analysis may be confounded by overlapping viral transcripts. For Figure 5F, as UL145 transcripts do not overlap with transcripts from the neighboring UL144 and UL146 genes, the sequences detected by RNAseq could be reliably ascribed to UL145 (Sun et al., 2007).

#### Statistical Analysis

The exact value of n within figures is indicated in the respective figure legends, and refers to the number of biological replicates. Blinding or sample-size estimation was not appropriate for this study. There were no inclusion criteria and no data was excluded.

Figures 1, S2, S3, and S5. The inhibitor experiments were performed in single replicates at each of 12, 18 and 24 h after infection. Protein ‘rescue ratios’ were approximately normally distributed (Figure S1B). The method of significance A was used to estimate the p-value that each ratio was significantly different to 1 (Cox and Mann, 2008). Values were calculated and corrected for multiple hypothesis testing using the method of Benjamini-Hochberg in Perseus version 1.5.1.6 (Cox and Mann, 2008). A corrected p-value <0.01 was considered statistically significant.

Figures 2, S2, S3, and S5. The pSILAC experiments were performed in single replicates at each of 6 and 18 h after infection. With respect to protein concentration, protein degradation typically follows first order kinetics whereas protein synthesis is a zero-order process. For pSILAC data, the rate of protein decline in mock- and HCMV-infected samples was therefore estimated using exponential regression in Excel and the formula [protein] (t) = e^Kdeg x t^ where Kdeg is the rate constant for degradation, and should be negative for degraded proteins. A degradation ratio was calculated by rdeg = Kdeg_HCMV_/Kdeg_mock_. In cases where this ratio could not be calculated because Kdeg_mock_ was greater than 0, a fold change (FC_HCMV_) in protein abundance in the HCMV-infected sample at 6 h (6 h pSILAC experiment) or 18 h (18 h pSILAC experiment) was instead used, defined by FC_HCMV_ = 1/e^Kdeg(HCMV) x t^. Protein half-life was estimated by t_1/2_ = ln(0.5)/Kdeg. The corresponding rates of protein synthesis were estimated using linear regression in Excel and the formula [protein] (t) = Ksyn x t where Ksyn is the rate constant for synthesis. We determined if Kdeg_HCMV_ was significantly different to Kdeg_mock_, and if Ksyn_HCMV_ was significantly different to Ksyn_mock_ using an in-house script written in R (version 3.4.2). For each peptide, the difference in paired normalised signal:noise values at each of the five measured time points was calculated. A simple linear regression model without an intercept was fitted to paired difference data from all peptides for each protein, and a p-value calculated for the null hypothesis of the slope being zero. All p-values were corrected for multiple hypothesis testing using the method of Benjamini-Hochberg (Benjamini and Hochberg, 1995). A Benjamini-Hochberg-corrected p-value <0.05 was considered statistically significant.

For Figure 2E, all viral proteins were included where Ksyn_HCMV_ was significantly higher than Ksyn_mock_ at p<0.05. As the measurement of viral proteins in mock-infected samples was at the level of noise, the value of the ratio Ksyn_HCMV_/Ksyn_mock_ was not considered. All human proteins were included with Ksyn_HCMV_/Ksyn_mock_ >3 and p<0.05.

Figures 3, S2, S3, and S5. The WCL2 experiment was performed using single replicates collected at multiple time points as detailed in the figure, apart from the mock sample which was collected in biological duplicate (Weekes et al., 2014). The RNAseq experiment was performed in biological triplicate at 0, 24 and 72 h after infection. Mean and SEM were calculated for normalised RPKM values for each time point 0, 24, 72 h (n=3). Fold change at 24h was calculated from mean RPKM(24 h)/mean RPKM(0h). A similar value was calculated for 72 h data. A Benjamini-Hochberg corrected student’s t-test was used to estimate the p-value for the hypothesis that a given transcript was expressed significantly differently at 24 or 72 h compared to mock infection. For protein expression from experiment WCL2, fold change at time t was calculated from S:N (t) / S:N (0h). p-values that a given protein was expressed significantly differently at 24 or 72 h compared to mock infection were estimated using Benjamini-Hochberg-corrected significance A values (Cox and Mann, 2008). XLStat (Addinsoft) was used to calculate the summed distance of each protein from its cluster centroid (Figure S4), and k-means clustering was performed in Cluster 3.0 (Stanford University).

Figures 5 and S6. The block deletion screens were conducted in partial biological duplicate as detailed below. Two block viral gene-deletion screens Block1 and Block2 were conducted. For each protein in each screen, a mean (μ) and standard deviation (σ) of all normalised S:N values was calculated. In each case, the maximum (x) value was omitted. For example, for HLTF in Figure 5A, μ and σ were calculated using values for wt1, wt2, RL10-UL1, RL11-UL11, UL2-UL11, US1-US11, US18-US22, US29-US34A but not the maximum U_L_/*b’*. The formula z = (x – μ)/σ was then applied to calculate a z-score. Fold change (FC) compared to wild-type (wt) infection was calculated from normalised S:N values using FC = x/wt1. Because of the limits of multiplexing with TMT, block deletion viruses AD169 (U_L_/*b’*), ΔUS27-28, ΔUL13-20, ΔUS12-17 were only examined in a single screen. All other block deletion viruses were examined in both screens. For each experiment, a given protein was initially assigned to the block corresponding to the TMT channel with the maximum S:N. To combine results to assign an overall block to each protein:

##### For Proteins Assigned to Blocks Studied in Both Screens

If the protein was quantified in both screens and assigned to the same block, z-scores and fold changes were averaged. For example, HLA-A11 was assigned to the US1-US11 block in both screens (Figure 5A), so the average of the two z-scores (17.1 and 112.6) was used to give a combined z=64.8. If the protein was only quantified in one of the two screens, the block assignment, z-score and fold change from that screen were used (for example, for CXADR, Figure S6B). Otherwise, it was not considered possible to assign an overall gene block for that protein.

##### For Proteins Assigned to One of the U_L_/b’, US27-28, UL13-20, US12-17 Blocks, Which Were Only Examined in 1/2 Screens

If the protein was quantified in both screens, and assigned to one of U_L_/*b’*, US27-28, UL13-20, US12-17 in 1/2 screens, the assignment in the other screen would have been different as these blocks were not examined in duplicate. To assign an overall block, the following rule was employed: if the z-score of the block assignment in one screen was at least 3.5 higher than the z-score of the block assignment from the other screen, the z-score and fold change from the former screen were used. For example, ABCC1 was assigned to the U_L_/*b’* block in Block1 (z=5.44, FC=2.23) and the US29-US34A block in Block2 (z=1.87, FC=1.16). Since the difference in z-scores was 3.57, the data for Block1 was used.

To confidently assign proteins to viral blocks, stringent criteria were used with a combined z-score of >6 and FC>2 or sensitive criteria with a combined z-score of >5 and FC>1.5. ABCC1 was therefore assigned to the U_L_/*b’* block by sensitive criteria, in keeping with our previous data (Weekes et al., 2013).

Figure 6. SILAC immunoprecipitations shown in Figures 6B and 6E were performed in single replicates. p-values were estimated using Benjamini-Hochberg-corrected significance A from Perseus version 1.5.1.6 (Cox and Mann, 2008).

Figure 7. All experiments in this figure were performed in biological triplicate. p-values were estimated using a 2-way ANOVA (B) or a two-tailed t-test (D, E, F).

#### Pathway Analysis

The Database for Annotation, Visualisation and Integrated Discovery (DAVID) was used to determine pathway enrichment (Huang da et al., 2009). A given cluster was always searched against a background of all proteins quantified within the relevant experiment. For Figure 4A, DAVID analysis examined all proteins identified as degraded at any of the three time points for the inhibitor screen, proteins degraded in either of the two time courses for the pSILAC screen, or proteins degraded at either time point for the RNA/protein screen.

### Data and Software Availability

Unprocessed peptide data files for Figures 1, 2, 3, and 5 have been deposited to Mendeley Data and are available at http://dx.doi.org/10.17632/zkgmjzrcyk.1. These files include details of peptide sequence, redundancy, protein assignment raw unprocessed TMT reporter intensities and isolation specificity. RNAseq metadata, processed data and FASTQ files can be accessed via GEO: GSE111036. The mass spectrometry proteomics data have been deposited to the ProteomeXchange Consortium (http://www.proteomexchange.org/) via the partner repository PRIDE: PXD009945.

## Supplemental Information

Document S1. Figures S1–S7

Table S1. Interactive Spreadsheet of All Data in the Manuscript, Related to Figures 1–4 The “Plots” worksheet generates graphs for all the human and viral proteins and transcripts quantified, and enables easy visualization of statistics. The “Data” worksheet shows minimally annotated protein and transcript data, for which the only modifications are formatting, normalization, reassignment of 11 non-canonical HCMV ORFs, and combination of peptide data for HLA-B and HLA-C alleles into single combined results, as described in STAR Methods. The “Calcs” worksheet shows all derived values, and the “Lookup” worksheet is used to generate graphs shown in “Plots”.

Table S2. Proteins Identified by All Screens in the Manuscript, and the Combination of Screens, Related to Figures 1–4 Separate worksheets are shown for each time point and each inhibitor (MG132/Leupeptin screen), the pSILAC and RNA/protein screens. In each of these cases, “stringent” criteria were used to filter proteins (Figure S1C). Additional worksheets show data for each of the 7 clusters displayed in Figure 3B, and data for the medium- and high-confidence lists of degraded proteins.

Table S3. Analysis of Synthesized Proteins from the 18-hr pSILAC Screen, Related to Figure 2 (A) Human proteins with Ksyn_HCMV_/Ksyn_mock_ > 3 and p < 0.05. (B) Viral proteins with p < 0.05. (C) Enrichment of functional pathways among human proteins from Table S3A.

Table S4. Enrichment of Functional Pathways, Related to Figure 4 (A–C) Within human proteins identified using “stringent” criteria in each individual screen. (D–E) Within the medium- and high-confidence lists of degraded proteins.

Table S5. Details of Viral Gene Deletion Mutants, Related to Figure 5 (A) Block deletion mutants; (B) single viral gene-deletion mutants.

Table S6. Predicted Block of Viral Genes Targeting 251 Human Proteins, Related to Figure 5

Table S7. Further Details of Materials and Techniques Used in This Paper, Related to STAR Methods (A) Details of TMT labeling; (B) details of RAW mass spectrometry files; (C) oligonucleotides used in this study.

Data S1. Further Details of ORFs Identified from the Six-Frame Translation, Related to STAR Methods For each ORF, peptide sequences quantified are shown in red. Any methionine residues preceding the most N-terminal peptide sequence are shown in purple. Further details of the ORF's relation to canonical genes are given, including a prediction of whether the ORF is likely to encode a functional protein. For two ORFs that are 5′-terminal extensions of previously described polypeptides, the original polypeptide sequence is shown outlined in gray.

Document S2. Article plus Supplemental Information
