# Supplementary material for: High-Definition Analysis of Host Protein Stability during Human Cytomegalovirus Infection Reveals Antiviral Factors and Viral Evasion Mechanisms
Source: Cell Host Microbe. 2018 Sep 12;24(3):447–460.e11. doi: 10.1016/j.chom.2018.07.011 (PMC6146656; doi:10.1016/j.chom.2018.07.011)
Supplement: Document S1. Figures S1–S7 [file mmc1.pdf]

Cell Host & Microbe, Volume *24*

Supplemental Information

High-Definition Analysis of Host Protein Stability during Human Cytomegalovirus Infection Reveals Antiviral Factors and Viral Evasion Mechanisms

Katie Nightingale, Kai-Min Lin, Benjamin J. Ravenhill, Colin Davies, Luis Nobre, Ceri A. Fielding, Eva Ruckova, Alice Fletcher-Etherington, Lior Soday, Hester Nichols, Daniel Sugrue, Eddie C.Y. Wang, Pablo Moreno, Yagnesh Umrania, Edward L. Huttlin, Robin Antrobus, Andrew J. Davison, Gavin W.G. Wilkinson, Richard J. Stanton, Peter Tomasec, and Michael P. Weekes

Figure S1

A

	12h inhibitor	18h inhibitor	24h inhibitor	6h pSILAC: medium	6h pSILAC: heavy	18h pSILAC: medium	18h pSILAC: heavy	Block deletion 1	Block deletion 2	Total any experiment
**Human proteins**	8033	8566	8545	7090	1116	7181	3813	8545	8912	10308
**HCMV canonical proteins**	81	96	92	0	26	3	70	125	136	139
**HCMV non-canonical ORFs**	4	12	12	0	2	0	4	23	16	27
**HCMV 6-frame translation ORFs**	0	4	3	0	0	0	0	7	10	13

B

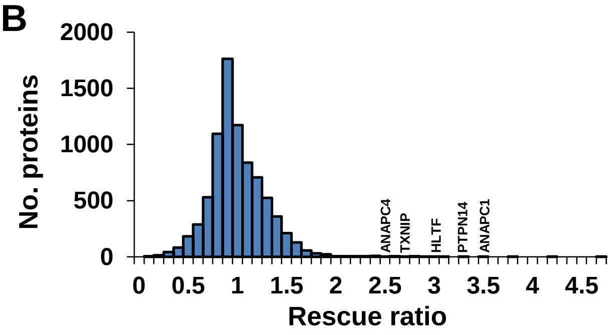

C

**1. Inhibitor screen**	**Stringent**	**Sensitive**
Fold down-regulation by HCMV (i)	>1.5	N/A
Rescue ratio (ii)	>1.5	>1.5
Rescue ratio p-value	<0.01	<0.01
**2. pSILAC screen**		
$Kdeg_{HCMV}/Kdeg_{mock}$ (iii) or FC_{HCMV} (iv)	>1.5	>1.5
p-value	<0.05	≤1
**3. RNA/protein screen**		
Protein fold down-regulation by HCMV (v)	>1.5	>1.5
RNA fold upregulation (vi)	>1	>1
Protein p-value	<0.05	≤1

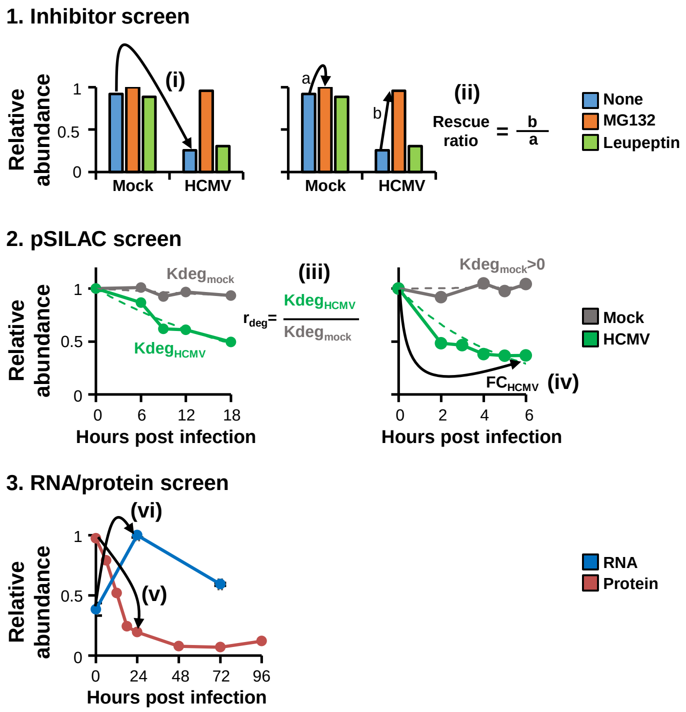

D

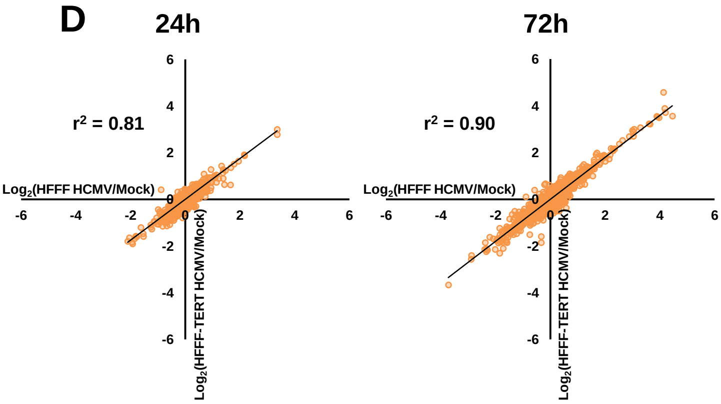

E

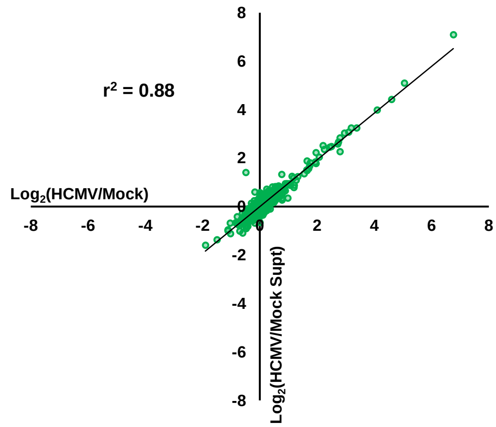

Figure S1, related to Figures 1-3.

(A) Numbers of human and viral proteins quantified in each experiment.

(B) Histogram illustrating approximate normal distribution of rescue ratios (see **Figure 1B**). Perseus software was used to estimate p-values for each ratio. The algorithm determined a ‘right’ and ‘left’ sided standard deviation (SD) and calculated a Significance A value for each fold change based on these SDs, which was used as an estimate of the p-value.

(C) Summary of ‘sensitive’ and ‘stringent’ criteria used in **Figures 1-3**. For consistency across all three screens, a 1.5-fold change cutoff value was used in each part of each criterion. This enabled detection of a wide range of proteins degraded with different kinetics, and facilitated identification of a shortlist of hits degraded with high confidence via the combination of the three screens.
For the <u>inhibitor screen</u>, two changes were examined: (i) downregulation of a given protein by HCMV, and (ii) rescue by inhibitor. Stringent criteria required >1.5-fold change for both (i) and (ii). Sensitive criteria did not require >1.5-fold downregulation by HCMV, to ensure inclusion of proteins that were initially upregulated during infection and yet nevertheless targeted for degradation, including interferon-stimulated proteins.
For the <u>pSILAC screen</u>, we required the comparative rate of degradation of a given protein during HCMV compared to mock infection rdeg = $Kdeg_{HCMV}$ / $Kdeg_{mock}$ >1.5 (iii). In some cases, rdeg could not be calculated accurately (i.e. $Kdeg_{mock}$ was >0). This generally occurred in the 6 h pSILAC screen, and was due to little change in the abundance of certain proteins in the mock sample over the short time interval studied. In these cases, we instead required FC_{HCMV} > 1.5 (iv), where FC_{HCMV} represented the fold downregulation in the HCMV-infected sample at 6 h (Experiment 2) or 18 h (Experiment 1), compared to time point 0.
For the <u>RNA/protein screen</u>, the fold downregulation of a given protein at time t was calculated from FCpro(t) = signal:noise (S:N) (0h) / S:N (t). FCpro(t) was required to be >1.5 at either 24 or 72 h (v), and transcript at the corresponding time point was required to be upregulated (vi).
For the inhibitor and RNA/protein screen, p-values were estimated using the method of Significance A, calculated in MaxQuant and corrected for multiple hypothesis testing using the method of Benjamini-Hochberg (Cox and Mann, 2008; Benjamini and Hochberg, 1995). Further details of p-value calculation for the pSILAC screen are given in Star Methods.

(D) Comparison of primary HFFF and immortalized HFFF-TERTs infected with HCMV or mock-infected for 24 or 72 h. 2348 proteins were quantified by ≥2 peptides. HCMV : mock ratios for each protein were highly correlated between cell types.

(E) Comparison of mock infection preparations. For all infections in this manuscript, we added the requisite volume of viral stock to DMEM, which was mixed and added to cells. Mock infections were performed identically but with the same volume of additional DMEM instead of viral stock. Since the last step in the preparation of our viral stocks was resuspension of the viral pellet in fresh DMEM, we did not anticipate any impact from adventitious factors carried along with the virus. To ensure this was actually the case, we generated two control media: (1) ‘Mock supt’: viral stock that had been centrifuged at 21,000 g to remove virus, then 190 ml of supernatant (equivalent of moi 3) mixed with 1 ml DMEM. This was filtered through a 0.22 mm sterile filter. (2) ‘Mock’: 190 ml DMEM mixed with a further 1 ml DMEM. We used a ‘singleshot’ analysis to measure 2753 proteins from whole cell lysates after 12 h infection of HFFF-TERTs with either mock or HCMV at moi 3. Data were highly correlated.

Figure S2

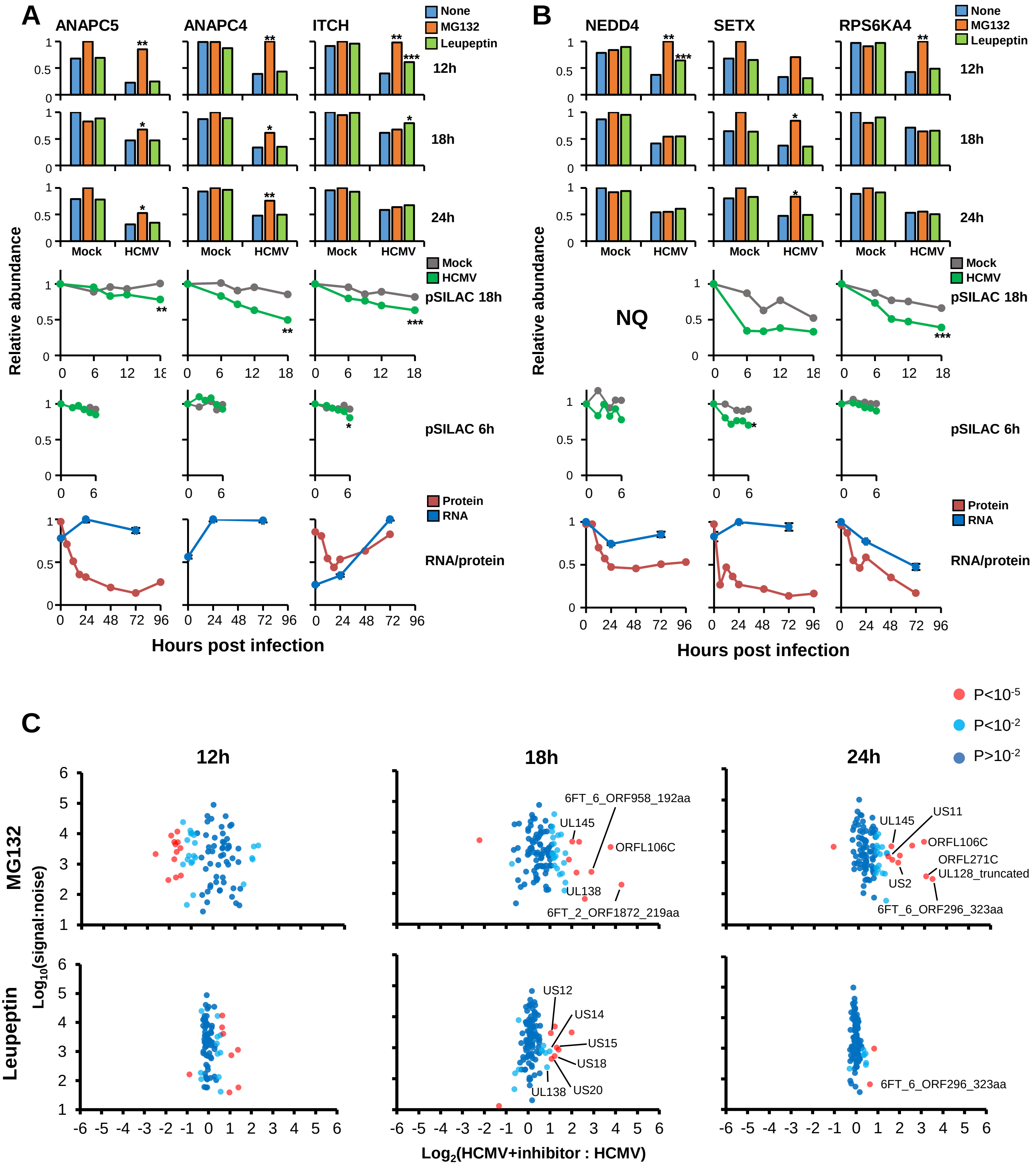
A
ANAPC5
ANAPC4
ITCH
None
MG132
Leupeptin
12h
18h
24h
Mock
HCMV
Relative abundance
pSILAC 18h
pSILAC 6h
Protein
RNA
RNA/protein
Hours post infection
B
NEDD4
SETX
RPS6KA4
NQ
C
P<10^-5
P<10^-2
P>10^-2
12h
18h
24h
MG132
Leupeptin
Log10(signal:noise)
6FT_6_ORF958_192aa
UL145
ORFL106C
UL138
6FT_2_ORF1872_219aa
US11
ORFL271C
UL128_truncated
US2
6FT_6_ORF296_323aa
US12
US14
US15
US18
US20
Log2(HCMV+inhibitor : HCMV)

Figure S2, related to Figures 1-3. Further examples of results from all three screens, and rescue of viral proteins by MG132 or Leupeptin.

(A) Further examples of proteins known to be targeted for proteasomal degradation by HCMV (see also **Figure 1B**). ANAPC4 protein was quantified in the inhibitor and pSILAC screens but not in the RNA/protein screen. p-values and error bars are shown as described in **Figures 1-3** * $p<0.05$, **$p<0.005$, ***$p<0.0005$.

(B) Novel observations of proteins found to be degraded by the screens. NQ – not quantified. p-values and error bars are shown as described in **Figures 1-3** * $p<0.05$, **$p<0.005$, ***$p<0.0005$.

(C) Scatter plot of viral proteins quantified in the inhibitor-based screens. Benjamini-Hochberg-corrected significance A was used to estimate p-values (Cox and Mann, 2008).

Figure S3

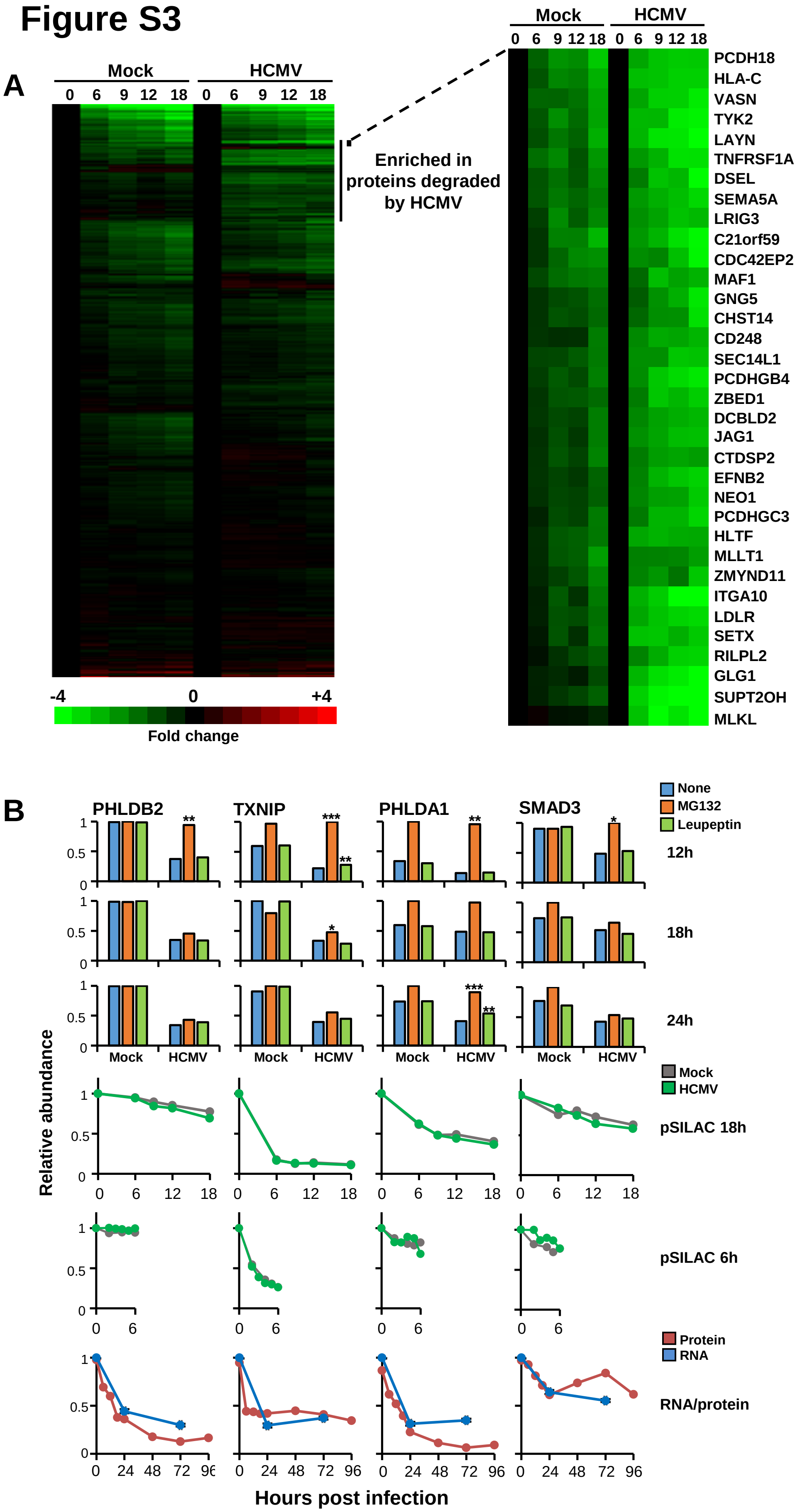
A
Mock
HCMV
0 6 9 12 18 0 6 9 12 18
Enriched in proteins degraded by HCMV
-4
0
+4
Fold change
PCDH18
HLA-C
VASN
TYK2
LAYN
TNFRSF1A
DSEL
SEMA5A
LRIG3
C21orf59
CDC42EP2
MAF1
GNG5
CHST14
CD248
SEC14L1
PCDHGB4
ZBED1
DCBLD2
JAG1
CTDSP2
EFNB2
NEO1
PCDHGC3
HLTF
MLLT1
ZMYND11
ITGA10
LDLR
SETX
RILPL2
GLG1
SUPT2OH
MLKL
B
PHLDB2
TXNIP
PHLDA1
SMAD3
None
MG132
Leupeptin
12h
18h
24h
Mock
HCMV
pSILAC 18h
pSILAC 6h
Protein
RNA
RNA/protein
Relative abundance
Hours post infection

Figure S3, related to Figures 1-3. Overview of data from the pulsed SILAC screens.

(A) Hierarchical cluster analysis of all medium-labelled proteins quantified in the 18 h pSILAC screen. The highlighted cluster is enriched in proteins exhibiting increased degradation in HCMV-infected cells compared to mock-infected cells. An enlargement of a subcluster is shown in the right panel, which included multiple proteins demonstrated to be degraded in other screens used in this study.

(B) Examples of proteins identified by the inhibitor-based screen that appeared inconsistent by pSILAC. p-values and error bars are shown as described in **Figures 1-3** * $p<0.05$, ** $p<0.005$, *** $p<0.0005$.

Figure S4

A

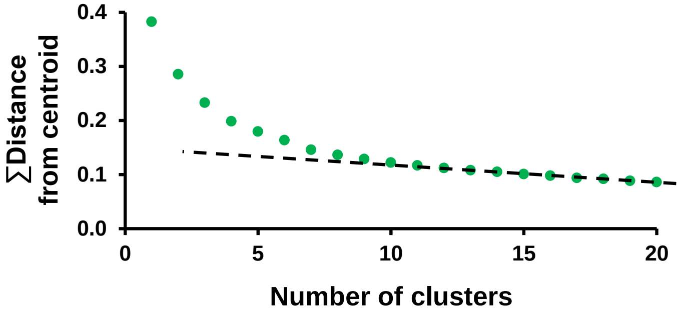

Figure S4, related to Figure 3. K-means analysis of human protein / RNA expression patterns during HCMV infection.

(A) Number of different classes of combined protein / transcript abundance. K-means clustering with 1-20 classes was used to assess the summed distance of each protein from its cluster centroid. While this summed distance necessarily becomes smaller as more clusters are added, the rate of decline decreases with each added group, eventually settling at a fairly constant rate of decline that reflects over-fitting; clusters added prior to this point reflect underlying structure in the temporal protein data, while clusters subsequently added through over-fitting are not informative. The point of inflexion fell between seven and nine classes, suggesting that there are at least seven distinct classes of temporal protein / RNA expression.

Figure S5

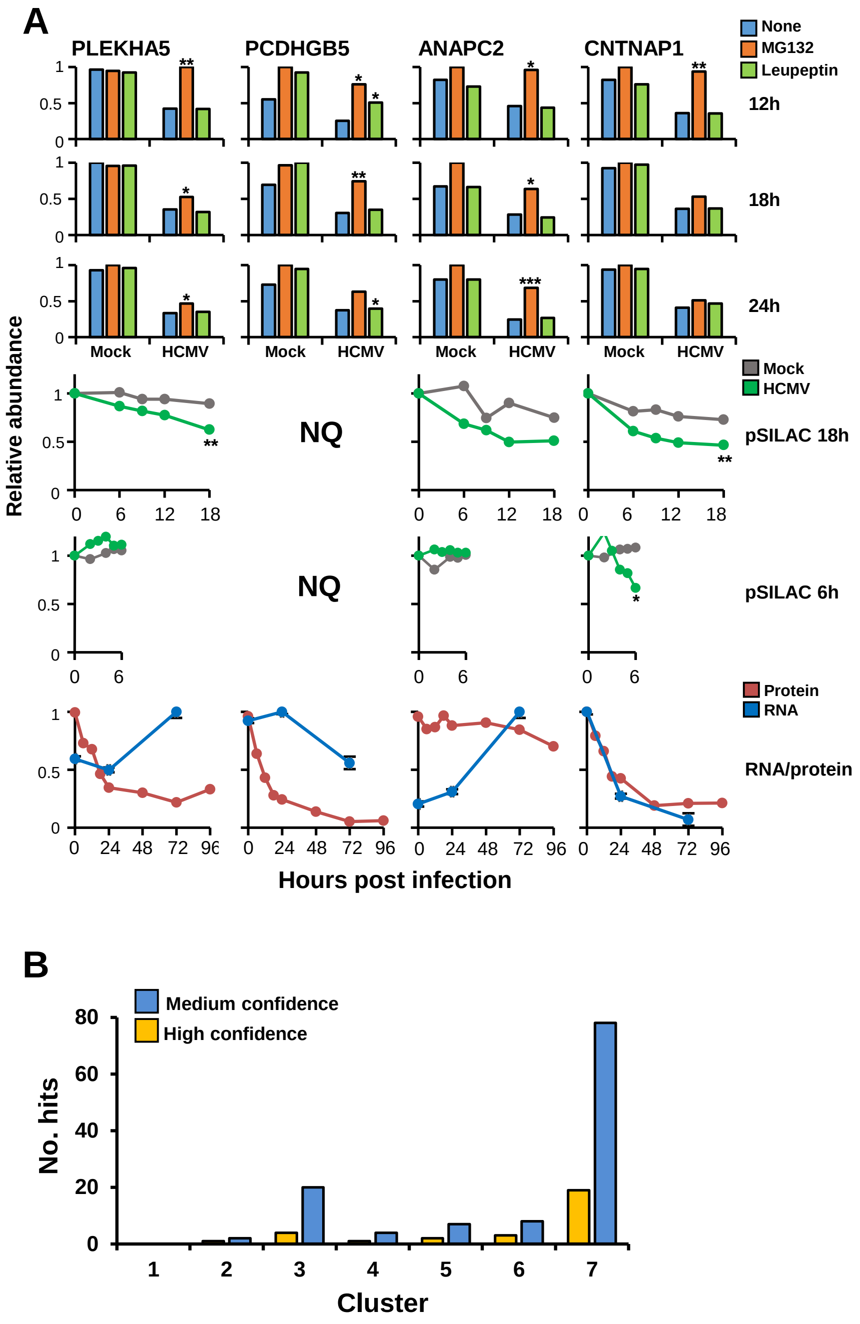

Figure S5, related to Figure 4. Overlap between all three screens.

(A) Examples of degraded proteins from DAVID analysis of data from individual screens. p-values and error bars are shown as described in **Figures 1-3** * $p<0.05$, ** $p<0.005$, *** $p<0.0005$.

(B) Numbers of proteins from the ‘medium confidence’ and ‘high confidence’ shortlists appearing in each cluster in **Figure 3B**. The majority of proteins from each list were found in cluster 7.

Figure S6

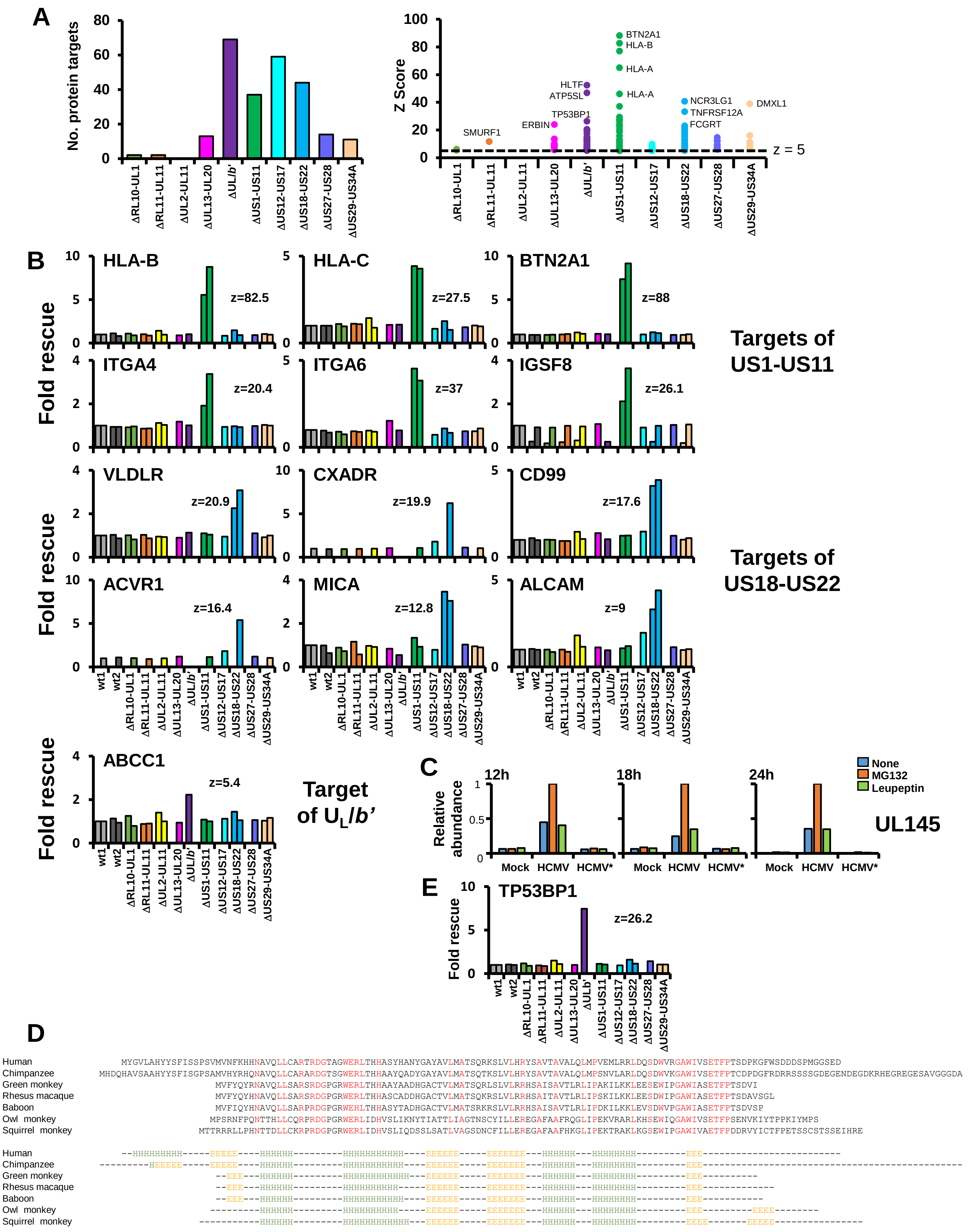
A
No. protein targets
Z Score
BTN2A1
HLA-B
HLA-A
HLA-A
HLTF
ATP5SL
TP53BP1
ERBIN
SMURF1
NCR3LG1
TNFRSF12A
FCGRT
DMXL1
z = 5
ΔRL10-UL1
ΔRL11-UL11
ΔUL2-UL11
ΔUL13-UL20
ΔUL/b'
ΔUS1-US11
ΔUS12-US17
ΔUS18-US22
ΔUS27-US28
ΔUS29-US34A
B
Fold rescue
HLA-B z=82.5
HLA-C z=27.5
BTN2A1 z=88
ITGA4 z=20.4
ITGA6 z=37
IGSF8 z=26.1
Targets of US1-US11
VLDLR z=20.9
CXADR z=19.9
CD99 z=17.6
ACVR1 z=16.4
MICA z=12.8
ALCAM z=9
Targets of US18-US22
wt1
wt2
ABCC1 z=5.4
Target of UL/b'
C
Relative abundance
12h
18h
24h
None
MG132
Leupeptin
UL145
Mock
HCMV
HCMV*
E
TP53BP1 z=26.2
D
Human
Chimpanzee
Green monkey
Rhesus macaque
Baboon
Owl monkey
Squirrel monkey

Figure S6, related to Figure 5. Validation of the proteomic screen of viral block deletion mutants.

(A) Numbers of human proteins that are targeted by each block using sensitive scoring (z-score of >5 and FC >1.5, left panel). For each block, z-scores of all proteins that passed sensitive scoring criteria are shown (right panel). For DUS12-US17, multiple proteins had similar z-scores close to 5.

(B) Further examples of known targets of the US1-US11, US18-US22 and U_L/*b'* blocks. Only single bars are shown for AD169 (U_L/*b'*), DUS27-US28, DUL13-UL20 and DUS12-US17 mutants, as these were assessed only in a single screen due to the multiplexing limits of TMT. Peptides from CXADR and ACVR1 were quantified in only one of the two screens.

(C) UL145 was rescued throughout the MG132 proteomic timecourse studied and was not quantified above mock levels from the HCMV* sample.

(D) Amino acid sequence alignment of UL145 orthologues in primate cytomegaloviruses, derived using Clustal Omega (http://www.ebi.ac.uk/Tools/msa/clustalo/). Fully conserved residues are in red font. The lower panel shows secondary structure predictions by Jpred 4 (http://www.compbio.dundee.ac.uk/jpred4). H, helical regions; E, extended regions.

(E) The U_L/*b'* block targets TP53BP1.

Figure S7

A

	12h inhibitor		WCL2	
	PD	MassPike	PD	MassPike
Quantified proteins	7641	8033	7434	7688
Overlap full Uniprot term	7114		6920	
Overlap 6-digit Uniprot term	7396		7163	

B

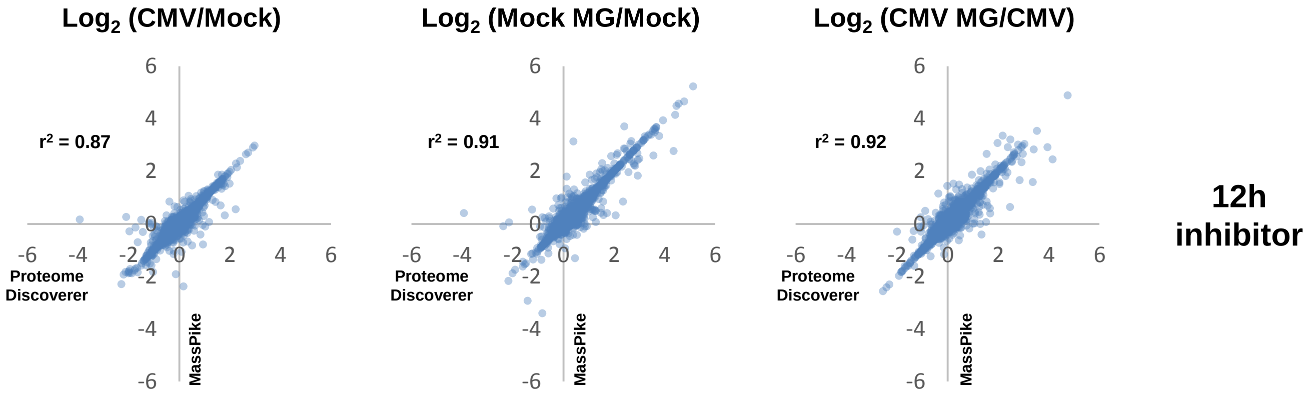

C

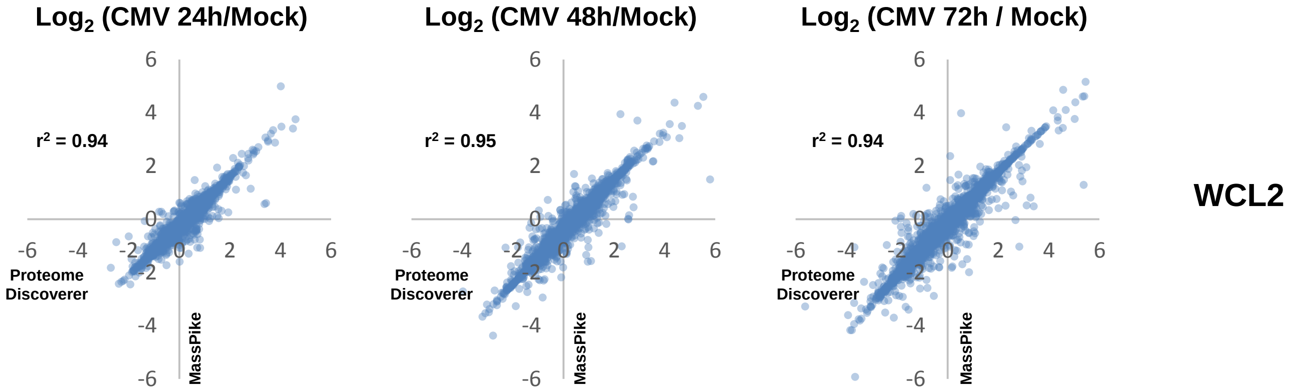

Figure S7, related to Star Methods. Comparison of data analysis using MassPike and Proteome Discoverer (PD).

(A) Comparison of numbers of human proteins quantified by both software packages. We used PD to analyse the 12 h experiment from the inhibitor screen (**Figure 1**) and the whole cell lysate timecourse WCL2 from the RNA/protein screen (**Figure 3**). More proteins were quantified by MassPike software in each case, despite including peptides identified by both "high" and "medium" confidence (false discovery rate (FDR) 1% and 5%) for PD, but only using a peptide-level FDR of 1% for MassPike. The overlap between datasets from each software was assessed using complete Uniprot terms that included isoform descriptions (e.g. Q9H0V9-2), or 6-digit Uniprot terms (e.g. Q9H0V9).

(B) Comparison of TMT data for all human proteins quantified by both software (identified using complete Uniprot terms) for the 12h inhibitor experiment.

(C) Comparison of TMT data for all human proteins quantified by both software (identified using complete Uniprot terms) for the WCL2 timecourse experiment.
