# Supplementary material for: High-Definition Analysis of Host Protein Stability during Human Cytomegalovirus Infection Reveals Antiviral Factors and Viral Evasion Mechanisms
Source: Cell Host Microbe. 2018 Sep 12;24(3):447–460.e11. doi: 10.1016/j.chom.2018.07.011 (PMC6146656; doi:10.1016/j.chom.2018.07.011)
Supplement: Data S1. Further Details of ORFs Identified from the Six-Frame Translation, Related to STAR Methods — For each ORF, peptide sequences quantified are shown in red. Any methionine residues preceding the most N-terminal peptide sequence are shown in purple. Further details of the ORF's relation to canonical genes are given, including a prediction of whether the ORF is likely to encode a functional protein. For two ORFs that are 5′-terminal extensions of previously described polypeptides, the original polypeptide sequence is shown outlined in gray. [file mmc9.pdf]

EXPRESSED NOVEL ORFS FROM A SIX-FRAME TRANSLATION OF THE HCMV STRAIN MERLIN GENOME

1. **6FT_1_ORF2139_186aa 0 unique peptides, 6 total peptides**

DQRVVRQVVDEHAEPGAQRQVQSVLRAQTLGMAGLVAHRRVGRGVHLVHHGRHQHDPRVPRRQLTQVRRA
HAVHLAARRHPRVESVRPSERQQRARVALRPLAHGAARAVGQKRRAPHGAHGLVFQLRQRLALHRNLRQQ
QALRAVPKVAVVAATRRGARRPAGFYPVDAVARRRRRRSTRTSARC

Coordinates: 234079-234639
Location: completely antiparallel to TRS1
Function: 18/244 genomes contain a stop codon before the M codon (3 types); 23/242 genomes contain stop codons after the M codon (6 types with 1, 2 types with 2); **very unlikely** to encode a functional protein.

2. **6FT_1_ORF94_49aa 1 unique peptide, 1 total peptide**

KHHSNYHDKFQKNNVKYCYHYTLVSNDWQSRFYCSVVNHNISNQPQSYE

Coordinates: 11326-11475
Location: completely parallel to RL13
Function: present in only one RL13 genotype; 3/22 genomes contain a stop codon (1 type); **very unlikely** to encode a functional protein.

3. **6FT_2_ORF1872_219aa 1 unique peptide, 1 total peptide**

RPLNDEAPKSTDPCLSCLKNVTTNASLPKATRSSRCARPNPRMTTKILPTTNCRRAHPRNISRQTNLRAY
AKRAPAHLRCRQSNGTVPPTEKFLRPDSRSIIKDRPDRQDRHRPNSGIEPRPISRKICETCTSICVHLRA
TGHGRQHLRGRKNVKHTPLTTFIIESLSSPYVVRTCCRSSLAAKPVLPSRVAASSVAPKMGIWVFSRVAS
STTAADRRH
Coordinates: 198026-198685
Location: 5' end parallel to IRS1 and 3' end antiparallel to US1
Function: 18/240 genomes contain a stop codon after the M codon (3 types); **very unlikely** to encode a functional protein.

4. **6FT_4_ORF330_368aa 1 unique peptide, 1 total peptide**

FVEIQPGFVVVNCAERAPRLIIVSERIADACLSTERWGHRCSVRFGLARWGWRLFHEPRRFPNLIETDIL
FGQLTLTLCHDGGEVETESSSVRKTKCDGIMYGVTALFAKHELPLPVRLARPQRGARVQVQRVPARPLPL
LAVQLGQQPPRGLGGGRRLAPQRQRAQHARAQKVRQLRRQRVHVPVQRAVPEHGAQLVQHHHQHGARHGP
RRRRTRHRRRTHHHRRRRRCCRCRRRRVRPDHRVRVRVWHANRAPAGASVRLRRYSHSRPHGSCHRARRV
PAGGLRLRAARRWRRRRPRRRPRRATQPPRGQHRAQRQPAAAQTLVQVPTHGRQQRRGVRRRRERLVLRQ
LLPRAQQHHRLQSPSRRL

Coordinates: 197967-196864
Location: completely antiparallel to IRS1
Function: 183/240 genomes contain stop codons (8 types with 1, 18 types with 2, 7 types with 3, 1 type with 4 and 1 type with 6); 12/242 genomes have an M codon (3 types, among which Merlin is unique); **very unlikely** to encode a functional protein.

5. **6FT_5_ORF380_169aa 2 unique peptides, 2 total peptides**

PRPHHATLRNSDATGDAQPARRTLLHAGVRSLLAGRPDLVADALASTESRLRDVPRFSTQLLLYRPRRRR
GLLETRMSGRGTRPLLDSDTALPAPRRLLPFAATPAEIFTGDASTAGRHRHRDVSLGHSGNRGIFGILEY
FHRMFPMLPVLPAVLWTLVPHAGKGTTRR

Coordinates: 184966-184457
Location: completely parallel to UL141
Function: 13/243 genomes contain a stop codon after the M codon (3 types); **very unlikely** to encode a functional protein.

6. **6FT_5_ORF5_488aa** **0 unique peptides, 3 total peptides**

RQQQQLLARPGAPVAVSDSGGAPGADGAGIPGHGRRVARGVSGQQHGPRHAQVVAARRGHAAAARTAVRI
LRAGDAAQPDERRGRDGPASAVAAGRVDRAGGDRGARGGPRGRPDGGRQGRPSRGSVRAGRTVPGAGRRV
PRVRLRPGEQHADPSGARRGRVVSARRGRGGAAVPLQPAGRGHPARDAAASAGAPTDVAARRGGDGARTG
AAPAVGRHDGGTTDAGKATAADGAAGRVAGAGAVRAVRVGAAPRVAADGRASAPEPASVLRLAGAGRGAA
RAVAGLRGGAGDGDDVAASGERHGDGGRRRGRAVRDGRSRGVCCDYTSAAVRRRRRERDLRAKRGRARGG
RGGRDGGGSGSSGGADGDGGSDGDGGSGGVGDRAARHAGGGAGRAGRRVRVLPAGRARVPAGGGAVALSA
RGRAGRAGAGARVGARRRGRAAAAARAGPRAVGAAALGRAAPAPARRAVGARAARAVSFFATSGPRRG

Coordinates: 234538-233072
Location: completely parallel to TRS1 and almost entirely in TRS
Function:; 75/243 genomes contain stop codons (5 types with 1, 5 types with 2); the stop codons in 4 types occur after the first peptide and involve 7 genomes; **very unlikely** to encode a functional protein.

7. **6FT_6_ORF1748_180aa** **1 unique peptide, 1 total peptide**

RHLEVMWPLLKNNVVRTGRRYAVFQPRRFTPRPQHDAFGTKDDVRGFTVFSHAACGASLMDPLSPSRWEV
ALFPSSPPSLKDSCHLCAWTFGLAGPCAAWLSTRRELVGGFSRIIYIQNSAECWSVREMKRCCRICRWRS
TSSSTDLRSNPYPIRWCYCWTMFPPMYPVLLPTASPVPTP

Coordinates: 27374-26832
Location: antiparallel to part of UL21A
Function: 17/244 genomes contain a stop codon after the M codon(5 types) or frameshifts (2 types); **very unlikely** to encode a functional protein.

8. **6FT_6_ORF181_361aa** **4 unique peptides, 4 total peptides**

PRWCAIRRTSCTTSSMRVISRARSACTWTSCTSLSPCSTSCLPSPAAPTLLRRRSLRRSLPQQRRRPSSS
PNRRVRGVTTSPCPTRSLVYKRRVGAPQRLCAETVATMQAQEANALLLSRMEALEWFKKFTVWLRVYAIF
IFQLAFSFGLGSVFWLGFPQNRNFCVENYSFFLTVLVPIVCMFITYTLGNEHPSNATVLFIYLLANSLTA
AIFQMCSESRVLVGSYVMTLALFISFTGLAFLGGRDRRRWKCISCVYVVMLLSFLTLALLSDADWLQKIV
VTLCAFSISFFLGILAYDSLMVIFFCPPNQCIRHAVCLYLDSMAIFLTLLLMLSGPRWISLSDGAPLDNG
TLTAASTTGKS

Coordinates: 2154050-214320
Location: N-terminal extension of US20 (grey)
Function: 5/243 genomes contain a stop codon (3 types) in the extension; the extension is **very unlikely** to be functional.

9. **6FT_6_ORF183_264aa** **9 unique peptides, 9 total peptides**

NRARFHSSRACRHASCRPARMDRRGGSPLPREAGGMAARLRPRRLPAYRHRRPQRAQLVAHAATGGRAMR
AGTGRRPTVAFSSQPRRARRLPVPAATRSVDRPLSPQTPTPVGLLPAARASLSDLADVPAVAAGLCRRGS
SRPGGRSQLPGGLAFGLRSWRVSPAAVATVGPVSALRYGAGTLAAAGRGPVLSRFTRDDAVDGDADANSR
SVPDHLSEFFPGEFSALATPLCRKCGFIHRHVPSSEALVVEPMTTGRIQGAATV

Coordinates: 214310-213516
Location: completely parallel to US19, with a short extension at each end
Function:; 0/244 genomes contain stop codons or frameshifts; BLASTP yielded no hits; equivalent ORF present in chimpanzee cytomegalovirus (CCMV) but not other cytomegaloviruses; this ORF, the corresponding part of US19 (from shortly after the start codon to the stop codon) and the corresponding irrelevant reading frame (regardless of stop codons) are 41, 49 and 38% identical, respectively, in HCMV and CCMV; **unlikely** to encode a functional protein.

10. **6FT_6_ORF296_323aa** **2 unique peptides, 2 total peptides**

RSVMTVVRSRPRVRASERRNATGSCMGSPRCSRNMNSHCPCGSRAHSAARGCRCSASQRGRSHCSRSSSG
SSRRAASAAGADSRPSASAPSTPARRKCDSSAASGYTCPSSGQYPNTAPSSSSTTTSMARGTVPDAAGPA
IAVGPTITVGAAAAAAAAAVASAPTTACASAFGTQIALPPAPPYGCGGIVTADPTAPAIAHGASPPAASV
SVPLAAGGDVVPVAGPAAQPSHRAGSTAPSASQPQHRRWFRCRRTAVSSDAGCGADANGSYCASSCHAPS
STIGCSRLPGVCSATVVPAHRRRSSRPSAVASSARSNVCRSAG

Coordinates: 197738-196767
Location: completely parallel to IRS1
Function: 86/242 genomes lack the first M codon but have the second; 7/242 genomes contain a stop codon (3 types); **very unlikely** to encode a functional protein.

11. **6FT_6_ORF305_233aa** **1 unique peptide, 1 total peptides**

RLHPPASTRNAAYSYAHTLSLHSDMLLRLCTAATSTSGHRHLPPHMAHVLRRPASNVVCSQHGAFFLARH
LHRTPSAAFAVASTREHTPQHALSPPRHSLLGFHTGFAHPAPGCQPQTFKDHVRDDRSHQFSNGLARQVR
QAGSRRRPSTPSTPRLAVAPPLPALPRPTRHFQQSHIPEGFLERRAPQTLRRDDIFVVVHHLHTGDYGLA
VAVRGTICRTQEKRNTNRGWVGR

Coordinates: 194303-193601
Location: completely parallel to UL150 and partly antiparallel to UL150A
Function: various frameshifts in region upstream from first M codon (including a G tract), analysed sequence after the first M codon; first M codon is present in all genomes, second M codon is not; 0/243 genomes contain stop codons; BLASTP yielded no hits other than Toledo UL151; corresponding ORF not present in CCMV; **unlikely** to encode a functional protein.

12. **6FT_6_ORF958_192aa** **1 unique peptides, 1 total peptides**

SRRYAGAAARDATPADGYPRTREPALADPGVAVHARLDAMPPRRQSAAGAAHVLYGQRGGERVGQRAQPH
GPKHLPQPRAHVDLCVRAAAQDAEHPQHQRAPLPVGGRAQTPTPARSRRCYSRVGQADVAGASHGLGTGL
DASAEPVERARRLLHVSVRVSHQGRGTAARGVRARAGLLHGEHARNQDAGDR

Coordinates: 122259-121680
Location: completely parallel to UL83
Function: 114/244 genomes contain stop codons after the M codon (2 types with 1, 1 type with 2); **very unlikely** to encode a functional protein.

13. **6FT_6_ORF1202_676aa** **4 unique peptides, 26 total peptides**

TPAKTRLAASAGAAGAAGAAGYCRPNAAGCNRQQTPRQRPPQPRASQQQQQSQPPWPLFRPQQQQQQP
PTQQHQATPYQLPPQQRRRTASHHQQQQPRRLAPRHQRQRPPPRWQTPTFASAPGPPEEGEECQTQPVI
SEPPSPEAEEPAAAVVEEVAPQAAATASGAEPASSTTSLYINVNVSRHSERPASYLCTDSMSLAGARPDD
SVSYVSESSHGDEFVTETMRSVFEMQRIRHGAGVSKVLRSERVTGGVHAVQEKRGGYSVSVPEDLPGGGG
AESYAEFAADSLSGDAAEGAARGYGFAGSGADGLLAPAGPGGLLPYRPPFGSLRSPSHRGAPVYGQRPFQ
RQSGHTQRHRALPVQNELRVRDPGAGGRPGGLRAAAGAPPSPMRHLAGGASGSVRRRDDMRPMLRGADHH
PESGPLAEQAAAGLAVQPYSGPPSVKPVRCEYPDGGAGPAGPDNAHPPLGWSPFGPQKPILFFIGLPQLH
PGGGGGAEGVQPVYGYTGTNIFLVGFYLLVPYLGGYRQAGGHHHPAAKCVSPAVPGAHERHQSPSVRGGR
GGHLRARGKGVGRGGAHVRGVGLCRPQQDHRPHHIPQHSSFRGQPGVQQAPRKQRDVHQNQAYSRGDSTS
AARWHGGRRPRGRGYSPAWTGGDVGDGYDFDDGQQQQQQQYSQSEE

Coordinates: 87962-85932
Location: parallel to 3’ end of UL57 and 5’ end of UL56
Function: 1/244 genomes contains a stop codon (1 type, yellos) that does not affect ORF147C (grey), which is almost completely parallel to UL56; BLASTP yielded no hits; an ORF of similar length to ORF147C is present in cytomegaloviruses and muromegaloviruses, but not other betaherpesviruses; alignments shown below; the part of UL56 that overlaps ORFL147C is conserved similarly to the part that does not overlap, ORFL147C is generally less well conserved than UL56, and conserved regions in

ORFL147C tend to be conserved more strongly in UL56, all of which indicates that selection may not have operated on ORFL147C; nonetheless, **possibly** encodes a functional protein.

```
HCMV     MNLLQKLCVVCSKCNEYAMELECLKYCDPNVLLAESTPFKRNAAAIVYLYRKIY[illegible]
CCMV     MNLLQKLCVVCSKCNEYAMELECLKYCDPNVLLAECTPFKRNAAAIVYLYRKIY[illegible]
BCMV     MNLLQKLCVVCSKCNEYAMELECLKYCDPNVLLAESSPFKRNALAIAYLYRRIY[illegible]
SCMV     MNLLQKLCVVCSKCNEYAMELECLKYCDPSVLLAESSPFKRNALAIAYLYRRIY[illegible]
RhCMV    MNLLQKLCVVCSKCNEYAMELECLKYCDPSVLLAESSPFKKNALAIAYLYRRIY[illegible]
SMCMV    MNLLQKLCVVCSKCNEYAMELECLKYCDPSILLAESSPFKRNALAITRLYRRIY[illegible]
OMCMV    MNLLQKLCVVCSKCNEYAMELECLKYCDPNILLAESSPFKRNAVAITRLYRRIY[illegible]
         ***************************.:****.:***:** **. ***:**[illegible]

HCMV     MSLAGARPDDSVSYVSESSHGDEFVTETMRSVFEMQRIRHGAGVSKVLRSERVTGGVHAVQEKRGGYSVSVPE[illegible]
CCMV     MSLSGAAPGDAVSYATEQGTGDEFVAETLCSVFEMQRIRHGAGMSKILRSQRFAGGMHAVQEKRSGYRVSVQE[illegible]
BCMV     MSLSEHITEDAVCYSDEQNTGDEFVAETVRSVFQMQRIRHGTGVSKVLRSERVAGGVKSVQEERTGYRVSVPQ[illegible]
SCMV     MSLPEHIPEDAVCYSDDQNAGDEFVAETVRSVFQMQRIRHGTGVSKVLRSERVAGGVKSVQEERTGYRLSVPQ[illegible]
RhCMV    MSLSEQITEDAVCYADEQGAGDEFVAETVRSVFQMQRIRHGTGVSKVLRSERVTGGVKSVQEERTGYRLSVPQ[illegible]
SMCMV    MYGAGNA---ESSDAGYDSTGNEFVAETVRGVFEMQRIRHGTGVSKILRSKHFAGGVESVQEECVGYHATVSS[illegible]
OMCMV    MYGSGSG---EAPEAECNSTGNELVAETVRGVFEMQRIRHGTGVSKVLRSEHFAGGVQSVQEECGGYYAAVPT[illegible]
         *                 .. *:*:*:**: .**:*******:*:**:***::.:**:.:***:  **  :*[illegible]

HCMV     MNLLQKLCVVCSKCNEYAMELECLKYCDPNVLLAESTPFKRNAAAIVYLYRKIY[illegible]
CCMV     MNLLQKLCVVCSKCNEYAMELECLKYCDPNVLLAECTPFKRNAAAIVYLYRKIY[illegible]
BCMV     MNLLQKLCVVCSKCNEYAMELECLKYCDPNVLLAESSPFKRNALAIAYLYRRIY[illegible]
RhCMV    MNLLQKLCVVCSKCNEYAMELECLKYCDPSVLLAESSPFKKNALAIAYLYRRIY[illegible]
SCMV     MNLLQKLCVVCSKCNEYAMELECLKYCDPSVLLAESSPFKRNALAIAYLYRRIY[illegible]
OMCMV    MNLLQKLCVVCSKCNEYAMELECLKYCDPNILLAESSPFKRNAVAITRLYRRIY[illegible]
SMCMV    MNLLQKLCVVCSKCNEYAMELECLKYCDPSILLAESSPFKRNALAITRLYRRIY[illegible]
MCMV     MNTLQKLCVVCSKCNECAMDVECLKYCDPNIVSMDSTAFRRNGVMVIHLYRTLY[illegible]
RCMVM    MNTLQKLCVVCSKCNECAMDAECLKYCDPSIVLMDSTAFQRNGATVIHLYRTLY[illegible]
RCMVE    MNTLQKLCVVCSKCNECAMDAECLKYCDPNIVLMDSTAFKKNGLMVIHLYRTLY[illegible]
         ** ************* **: ********.::  :.: *::*.  :  *** :*[illegible]

HCMV     MSLAGARPDDSVSYVSESSHGDEFVTETMRSVFEMQRIRHGAGVSKVLRSERVTGGVHAVQEKRGGYSVSVPE[illegible]
CCMV     MSLSGAAPGDAVSYATEQGTGDEFVAETLCSVFEMQRIRHGAGMSKILRSQRFAGGMHAVQEKRSGYRVSVQE[illegible]
BCMV     MSLSEHITEDAVCYSDEQNTGDEFVAETVRSVFQMQRIRHGTGVSKVLRSERVAGGVKSVQEERTGYRVSVPQDLPGGGATEPYADLAAEPVPGDDFEGSVRGYGAAGPRPQGVLEEAGQNGV[illegible]
RhCMV    MSLSEQITEDAVCYADEQGAGDEFVAETVRSVFQMQRIRHGTGVSKVLRSERVTGGVKSVQEERTGYRLSVPQDLPGGAGAPEPYTDFAVEPVHGDDFEGSVRGYGAARPRSQGVLQEAGPGGV[illegible]
SCMV     MSLPEHIPEDAVCYSDDQNAGDEFVAETVRSVFQMQRIRHGTGVSKVLRSERVAGGVKSVQEERTGYRLSVPQDLPGGGAPEPYPDLAAEPVPGDDFEGPVRGYGAAGPRPQGVFEEAGSNGV[illegible]
SMCMV    MYGAGNA---ESSDAGYDSTGNEFVAETVRGVFEMQRIRHGTGVSKILRSKHFAGGVESVQEECVGYHATVSSDLSGVGETESCADVATDSVYGSESESFVRGYGNAGSGFNEVLAEARPNGV[illegible]
OMCMV    MYGSGSG---EAPEAECNSTGNELVAETVRGVFEMQRIRHGTGVSKVLRSEHFAGGVQSVQEECGGYYAAVPTHLSGAGEAEPCPDVVAQSVYGGQFAGPVRRYGNSGPGLKEVFAKPGPNGV[illegible]
MCMV     MYKRRGRGV-DLLARNVGKDGNEYVTKTVRRVFQMQRVRDGCGMSEVLRSEYCVDGFDGLQEERGHGDTFVSDPLSGAGLAERGADLGADPLHGDAAAGLVRYDEGDRHGFDGLWDASGPAAI[illegible]
RCMVM    myrrrgrgv~~~~~~~~~~~~EYVTKTVRRVFQMQRVRDGRGMSEVLRSEYRVDGFDGLPEERGDGDPSLPDPVPGFGGAEYGADLGLEPVPGDALGGFVPYDEGAGYGFNGFREAPGPGEV[illegible]
RCMVE    MYRRRGRGV-VGIHRVLGKDGHEYVTKTVRRVFQMQRVRDGCGMSEVLRSEYCVDGFDGFQEERVDGDPSVQNPVSCVSESEYCSDFGTDPLHADAFAGIVRYDEGDRYGVNGLCDAPEQGNI[illegible]
         *                 *  *  *    **  ***:*.* *:*::***:  ..*.... *:        :   :   .  *   :.  : :  ..   . .           . .        :[illegible]
```

UL56 above, ORFL147C below, with blocks arranged spatially to allow comparisons of conserved regions
Shown as cytomegalovirus alignments followed by cytomegalovirus alignments
Possible frameshift affecting 5’-end of RCMVM ORFL147C, indicated by tildes (~)
ORFL147C is generally less well conserved than UL56, and conserved regions in ORFL147C tend to be conserved more strongly in UL56
Equivalents to ORFL147C not present in members of other betaherpesvirus genera
